# The Locus Coeruleus‐Periaqueductal Gray GABAergic Projection Regulates Comorbid Pain and Depression

**DOI:** 10.1002/advs.202503739

**Published:** 2025-04-07

**Authors:** Yuan Gao, Xue Zhang, Xiao‐Juan Liu, Yi‐Ling Sun, Cui Yin, Dong‐Liang Tang, Cheng Xiao, Chunyi Zhou

**Affiliations:** ^1^ Jiangsu Province Key Laboratory of Anesthesiology School of Anesthesiology Xuzhou Medical University Xuzhou Jiangsu 221004 China; ^2^ Jiangsu Province Key Laboratory of Anesthesia and Analgesia Application Technology NMPA Key Laboratory for Research and Evaluation of Narcotic and Psychotropic Drugs School of Anesthesiology Xuzhou Medical University 209 Tonghshan Road Xuzhou Jiangsu 221004 China; ^3^ Department of Anesthesiology Nanjing Drum Tower Hospital Affiliated Hospital of Medical School, Nanjing University Nanjing Jiangsu 210008 China

**Keywords:** depression, GABAergic neuron, locus coeruleus, neuropathic pain, noradrenergic neuron, ventrolateral periaqueductal gray

## Abstract

The locus coeruleus (LC) noradrenergic (NA) neurons regulate pain and depression through their projections to various downstream nuclei. Although GABAergic (GABA) neurons in and near the LC (LC‐GABA neurons) provide inhibitory synaptic inputs to LC‐NA neurons, it remains unknown whether they are implicated in neuropathic pain and comorbid depression through LC‐NA neurons. This study demonstrates that LC‐GABA neurons respond to noxious stimuli with enhanced activity, and stimulation of these neurons elevates pain thresholds and exerts an anti‐depressant‐like effect in naive and neuropathic pain mice. Conversely, inhibition of LC‐GABA neurons leads to hyperalgesia and depression‐like behaviors in naive mice and exacerbates existing pain‐ and depression‐like behaviors in neuropathic pain mice. Although LC‐GABA neurons inhibit pain responses in LC‐NA neurons, they modulate pain thresholds and depression‐like behaviors in a manner independent of LC‐NA neurons. In contrast, the projection from LC‐GABA neurons to the ventrolateral periaqueductal gray (vlPAG) is enhanced, and stimulation of this projection mimics that of LC‐GABA neurons conferring analgesic‐ and antidepressant‐like effects. This study reveals the enhancement of the LC^GABA^‐vlPAG^GABA^ projection as a compensatory mechanism in neuropathic pain and suggests that further augmentation of this projection may be a therapeutic strategy for the treatment of comorbid neuropathic pain and depression.

## Graphical abstract

LC‐GABA neurons exhibit elevated activity to noxious and aversive stimuli in chronic pain, and their activation alleviates pain‐ and depression‐like behaviors. While LC‐GABA neurons target LC‐NA neurons and GABA and Glu neurons in the vlPAG, they modulate the pain responses of LC‐NA neurons but regulate pain‐ and depression‐like behaviors through their projections to vlPAG, rather than via LC‐NA neurons.

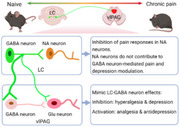

## 1 Introduction

Pain and depression are two common and debilitating conditions with a comorbidity rate ranging between 30% to 60%.^[^ ^1^ ^]^ Notably, pain is a significant risk factor for depression, and conversely, depression can worsen chronic pain.^[^ ^1^, ^2^ ^]^ Currently available pharmacological treatments for pain and depression are expensive and offer only modest benefits.^[^ ^1^, ^2^ ^]^ Therefore, improving our understanding of the neural circuits underlying comorbid pain and depression is essential for the development of more effective treatments.

Studies have identified specific brain circuits involved in pain and depression, including the locus coeruleus (LC), amygdala, insula, prefrontal cortex, anterior cingulate cortex, thalamus, and periaqueductal gray (PAG).^[^ ^1^, ^3^ ^]^ These circuits are linked to the emotional, sensory, and cognitive dimensions of pain. Among these structures, both human and animal studies have confirmed a connection between the LC and the comorbidity of pain and depression, with noradrenergic (NA) neurons playing an important role in this relationship.^[^ ^3b^ ^]^ Neural circuit dissection in rodents reveals that LC‐NA neurons provide endogenous inhibition of pain signals through their descending projections to the spinal cord, whereas to exert a depressant‐like effect by regulating cortical and subcortical nuclei, such as anterior cingulate cortex.^[^ ^3^, ^4^ ^]^ These circuit features compromise the potential of using the LC‐NA neurons as therapeutic targets to treat comorbid chronic pain and depression.

In addition to LC‐NA neurons, GABAergic (GABA) neurons in and around the LC have garnered increasing attention. Previous studies using ultrastructural microscopy and brain slice recordings have shown that LC‐NA neurons receive direct local GABA inputs.^[^ ^5^ ^]^ Moreover, LC‐GABA neurons receive inputs from numerous brain regions implicated in pain modulation and emotional processing,^[^ ^5a^ ^]^ including the prefrontal cortex, bed nucleus of the stria terminalis, lateral hypothalamus, PAG, paraventricular nucleus, and spinal trigeminal tract. This anatomical feature suggests that LC‐GABA neuronal activity may be influenced by various sensory and emotional stimuli. Additionally, LC‐GABA neurons projecting to LC‐NA neurons have been implicated in the regulation of physiological arousal by inhibiting LC‐NA neurons.^[^ ^5a^ ^]^ However, the contribution of LC‐GABA neurons to the comorbidity of pain and depression has not been elucidated.

In this study, we aimed to investigate the involvement of LC‐GABA neurons in hyperalgesia and depression‐like behaviors in neuropathic pain. Using in vivo fiber photometry, optogenetics, viral vector‐assisted tracing, and behavioral assessments, we found that activation of LC‐GABA neurons produced pain relief and antidepressant‐like effects in both normal and spared nerve injury (SNI) mice via projections to the ventrolateral periaqueductal gray (vlPAG). Notably, the contribution of LC‐NA neurons to the influence of LC‐GABA neurons on pain and depression‐like behaviors varied depending on behavioral phenotype and pain states. These findings establish the role of LC‐GABA neurons and their associated circuits in the regulation of pain and depression. The results suggest that the LC^GABA^‐vlPAG pathway may serve as a potential therapeutic target for the treatment of comorbid pain and depression.

## 2 Results

### 2.1 LC‐GABA Neurons Enhance Activity in Response to Nociceptive Stimulation

**Figure 1:**
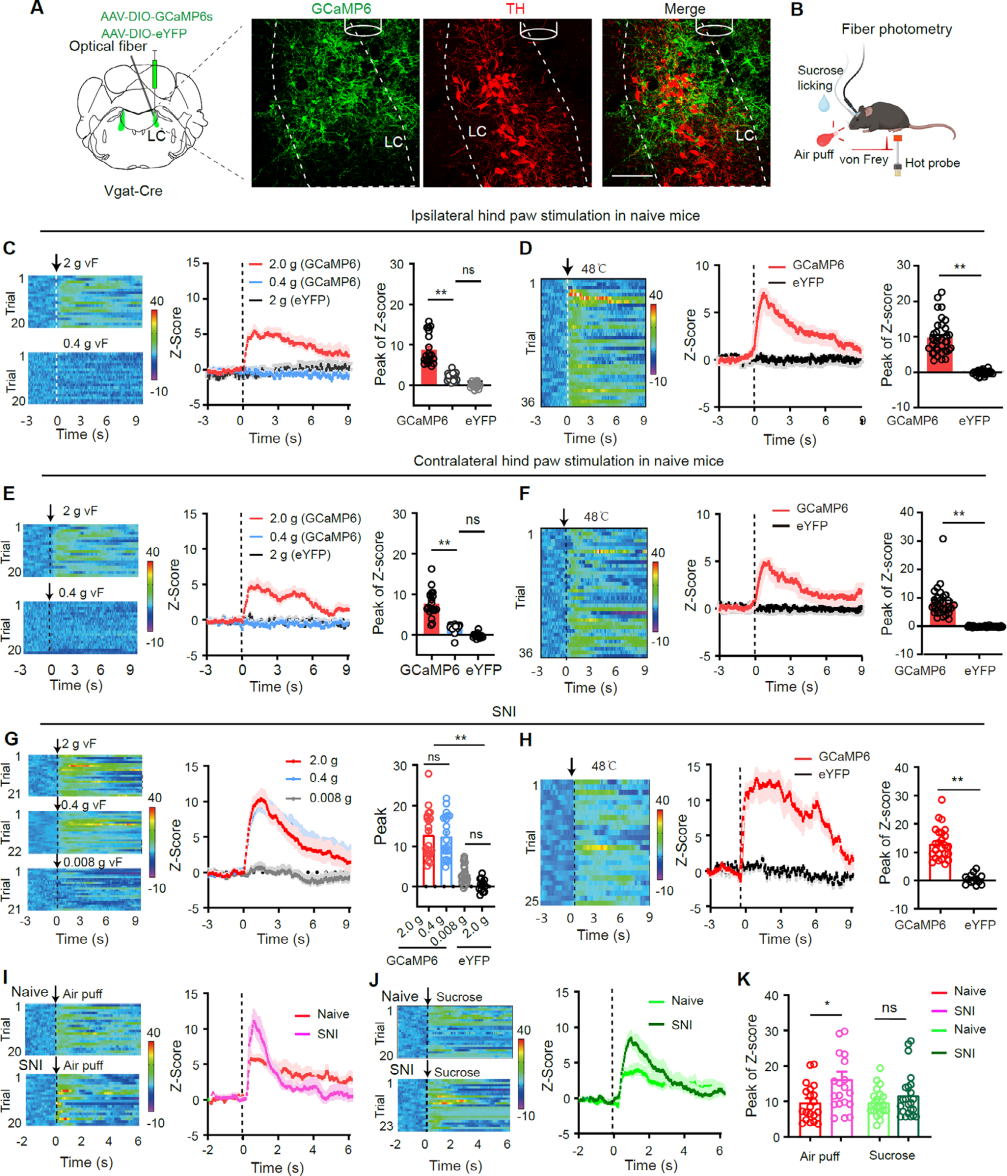
Locus Coeruleus GABAergic (LC‐GABA) neurons respond to pain‐like stimuli and emotional changes. A) Vgat‐Cre mice were injected with AAV‐EF1α‐DIO‐GCaMP6s or AAV‐EF1α‐DIO‐eYFP in the LC. Images of a coronal section show GCaMP6s expressing‐GABA neurons in and around the LC. The location of LC was identified by immunofluorescent staining with tyrosine hydroxylase (TH)‐antibody, which labels noradrenergic (NA) neurons. B) Schematic diagram for fiber photometry recordings of GCaMP6s‐ or eYFP‐labeled LC‐GABA neurons in response to various stimuli, including von Frey filament application and thermal stimulation on the hind paws, air puff on one side of the face, and licking of sucrose solution in freely moving mice. C–F) Heat maps (Left panels), averaged normalized traces (Middle panels), and summary (Right panels) showing changes in GCaMP6s and eYFP signals in LC‐GABA neurons in response to suprathreshold (2 g) or subthreshold (0.4 g) von Frey filament stimulation as well as thermal stimulation with a 48 °C iron block on either hind paw in naïve mice. *F*_(2, 54)_ = 73.04, *P* < 0.0001 for C); *t *= 10.88, *P* < 0.0001 for D); *F*_(2, 51)_ = 66.99, *P* < 0.0001 for E); *t *= 9.3, *P* < 0.0001 for F). G,H) Heat maps (Left panels), averaged normalized traces (Middle panels), and summary (Right panels) showing changes in GCaMP6s and eYFP signals in LC‐GABA neurons in response to suprathreshold (2 and 0.4 g) or subthreshold (0.008 g) von Frey filament stimulation as well as thermal stimulation on the hind paw of injured side in SNI mice. *F*_(3, 74)_ = 39.18, *P* < 0.0001 for G); *t *= 6.06, *P* < 0.0001 for H). I,J) Heat maps (Left panels), averaged normalized traces (Middle panels) showing changes in GCaMP6s signals in LC‐GABA neurons in response to air‐puff stimulation on one side of the face or sucrose licking behavior in naïve and SNI mice. K) Quantification of changes in GCaMP6s signals in panels I and J (Air puff: *t *= 2.55, *P* = 0.014; Sucrose licking: *t* = 1.12, *P* = 0.27). * *P* < 0.05, ** *P* < 0.01; One‐way ANOVAs with Tukey's post‐hoc analysis for panels C, E, and G; Unpaired *t*‐test for panels D, F, H, K; *n* = 5 mice in each group, and 4–5 trials were conducted from each mouse. Dashed lines indicate stimulus onset. Scale bars: 100 µm. Both the Z‐score and heat map color bars are in units of Δ*F*/*F*₀.

To visualize neural activity dynamics of LC‐GABA neurons in response to nociceptive and salient stimulations, we used in vivo fiber photometry to monitor their Ca^2+^ levels. AAVs encoding Cre‐dependent GCaMP6s or eYFP were injected into the LC of Vgat‐Cre mice (**Figure** **1**A). Three weeks after virus injection, GCaMP6 signals were recorded from LC‐GABA neurons in freely moving mice upon mechanical, thermal, aversive, and reward‐related stimulations (Figure 1A,B). In naive mice, suprathreshold (2 g) von Frey filament stimulation to either hind paw induced a strong increase in GCaMP6 signal (Figure 1C), while subthreshold (0.4 g) filament caused no significant signal changes (Figure 1E). Similarly, thermal stimulation with a 48 °C heating block elicited robust increases in GCaMP6 signal (Figure 1D,F). No changes in fluorescent signal were observed in eYFP control mice (Figure 1C–F), confirming the specificity of the GCaMP6 signal. Importantly, while no significant differences in peak signal levels were found upon stimulation on contralateral and ipsilateral hind paw (Mechanical response: *t *= 0.18, *P* = 0.86; thermal response: *t* = 1.25, *P* = 0.22; *n* = 36 repeated experiments from 5 mice) (Figure 1C–F). However, thermal stimulation induced significantly higher peak signals than von Frey stimulation (ipsilateral response: *t *= 3.40, *P *= 0.0011; contralateral response: *t* = 2.59, *P* = 0.012; *n* = 36 repeated experiments from 5 mice) (Figure 1C–F). These data suggest that LC‐GABA neurons respond to different types of sensory stimuli.

In SNI mice (Figure S1A,B, Supporting Information), GCaMP6 signal in LC‐GABA neurons was significantly enhanced in response to both suprathreshold (2 and 0.4 g) but not subthreshold (0.008 g) von Frey filament stimulation (Figure 1G). Thermal stimulation also elicited a significantly greater GCaMP6 signal in SNI mice (Figure 1H), compared to naïve mice (*t *= 2.43, *P* = 0.018, *n* = 25, 36 repeated experiments from 5 mice per group). These data indicate a heightened sensitivity to nociceptive stimuli in SNI mice.

We also examined the response of LC‐GABA neurons to aversive (air puff on one side of the face) and rewarding (sucrose‐licking behavior) stimuli (Figure 1I–K). Air puff stimulation significantly increased the GCaMP6 signal in both naïve and SNI mice, with a stronger response in SNI mice (Figure 1I,K). Sucrose licking elicited significant GCaMP6 signal changes in both groups with comparable magnitudes (Figure 1J,K).

These results suggest that LC‐GABA neurons are responsive to nociceptive and salient stimuli. Notably, in SNI mice, the aversive responses of LC‐GABA neurons were significantly enhanced, reflecting heightened sensitivity under neuropathic pain conditions.

### 2.2 LC‐GABA Neurons Bidirectionally Regulate Pain Thresholds and Depression‐Like Behaviors in Physiological Conditions

**Figure 2:**
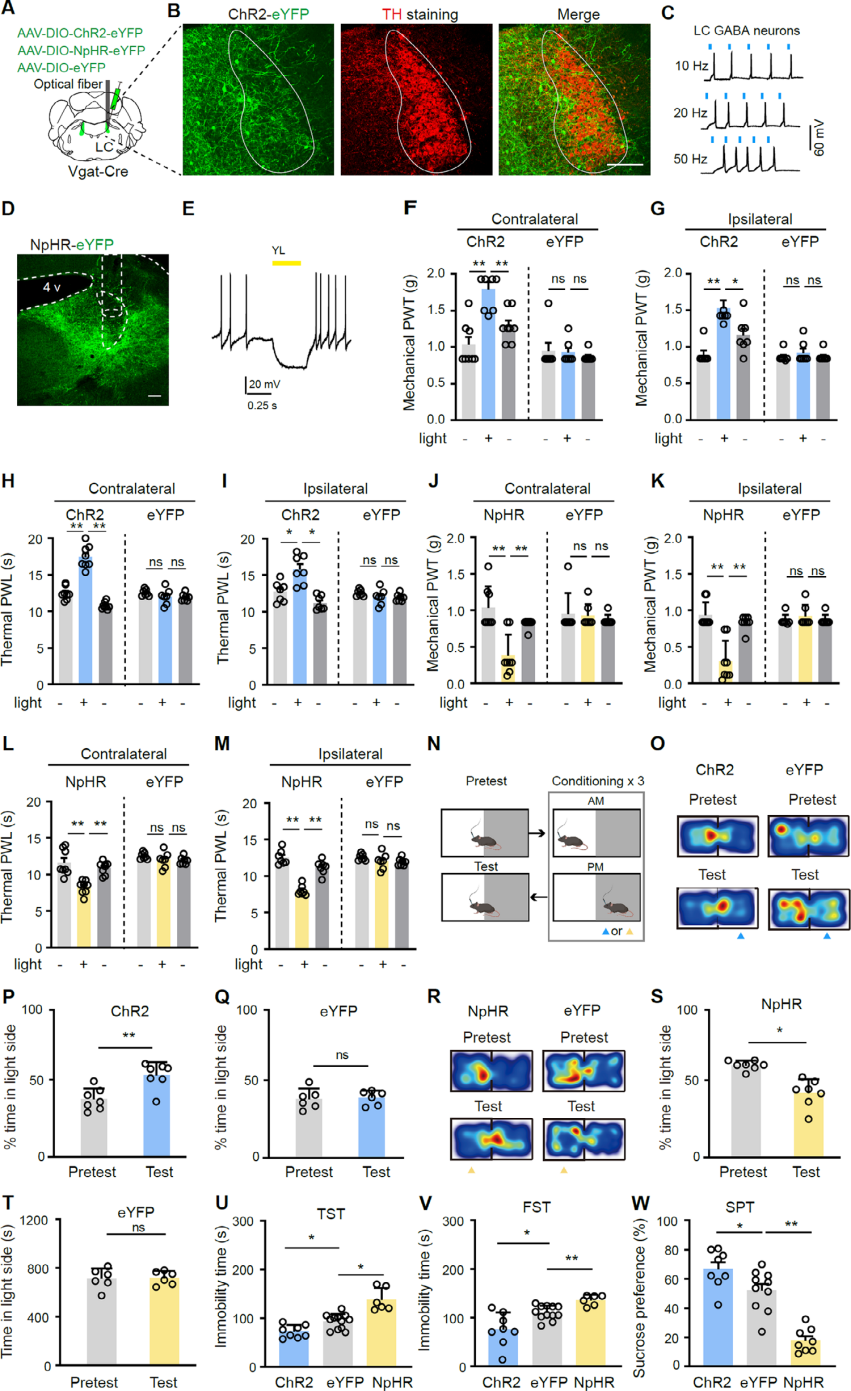
LC‐GABA neurons regulate pain thresholds and depression‐like behaviors in naïve mice. A) Schematic diagram illustrating virus injection and optical fiber placement for selective activation and inhibition of LC‐GABA neurons. B) Representative images from a coronal section showing ChR2 expression in the LC. The location of the LC was confirmed by immunohistochemistry staining with a TH‐antibody. C) Action potentials recorded in a ChR2‐positive LC neuron during blue light stimulation (473 nm, 5 ms, blue bars). D) Representative image of a coronal section showing NpHR expression in the LC. E) Voltage change recorded in an NpHR‐positive LC neuron during yellow light stimulation (589 nm, 500 ms, yellow bar). F–I) Mechanical PWT and thermal PWL in ChR2 and eYFP mice before, during, and after blue light stimulation (473 nm, 5 ms, 4 mW, 20 Hz, 1–2 min). *F*₍₁, ₁₃₎ = 23.99, *P* = 0.0003 for F); *F*₍₁, ₁₃₎ = 45.97, *P* < 0.0001 for G); *F*₍₁, ₁₃₎ = 23.38, *P* = 0.0003 for H); *F*₍₁, ₁₃₎ = 7.46, *P* = 0.018 for I). *n* = 8 in ChR2, *n* = 7 in eYFP. J–M) Mechanical PWT and thermal PWL in NpHR and eYFP mice before, during, and after yellow light stimulation (589 nm, 3 mW, 1–2 min). *F*₍₁, ₁₃₎ = 5.28, *P *= 0.039 for J); *F*₍₁, ₁₃₎ = 13.08, *P* = 0.0031 for K); *F*₍₁, ₁₃₎ = 21.79, *P* = 0.0004 for L); *F*₍₁, ₁₃₎ = 21.63, *P *= 0.0006 for M). *n* = 8 in NpHR, *n* = 7 in eYFP. N) Experimental setup for conditioned place preference or aversion. O–Q) Example heat maps (O) and quantification of percentage time spent in the blue‐light‐paired chamber during pretest and test sessions (P,Q) in ChR2 and eYFP mice. *t *= 3.76, *P* = 0.009 for P); *t* = 0.21, *P* = 0.84 for Q); *n* = 7 in ChR2, *n* = 6 in eYFP. R–T) Example heat maps (R) and quantification of percentage time spent in the yellow‐light‐paired chamber during pretest and test sessions (S,T) in NpHR and eYFP mice. *t* = 4.61, *P* = 0.004 for S); *t* = 0.12, *P* = 0.91 for T); *n* = 7 in NpHR, *n* = 6 in eYFP. U–W) Quantification of depression‐like behaviors in ChR2, NpHR, and eYFP mice. Immobility time in the tail suspension test (TST): *F*₍₂, ₂₃₎ = 12.98, *P* = 0.0002 for U); Immobility time in the forced swimming test (FST): *F*₍₂, ₂₃₎ = 26.41, *P* < 0.0001 for V); The sucrose preference test (SPT): *F*₍₂, ₂₃₎ = 33.78, *P* < 0.0001 for W). *n* = 8 in ChR2, *n* = 12 in eYFP, *n* = 6 in NpHR for panels U and V; *n* = 8 in ChR2, *n* = 10 in eYFP, *n* = 8 in NpHR for W. *P < 0.05, **P < 0.01; Two‐way repeated measures ANOVAs with Tukey's post‐hoc analysis for panels F–M; One‐way ANOVAs with Tukey's post‐hoc analysis for panels U–W; Two‐tailed unpaired *t*‐test for panels P, Q, S, and T. The *F*‐values reported indicate the effect of the group factor in the two‐way ANOVAs. Scale bars: 100 µm.

Based on the results from fiber photometry, we aimed to examine whether LC‐GABA neurons regulate pain‐like behaviors. We targeted LC‐GABA neurons for optogenetic stimulation and inhibition by unilaterally injecting Cre‐dependent viral vectors driving the expression of either the excitatory opsin ChR2 or the inhibitory opsin NpHR (or the fluorophore eYFP as control) into the LC of Vgat‐Cre mice (**Figure** **2**A,B,D). Patch‐clamp recording data confirmed that blue light stimulation (473 nm, 5 ms, 20 Hz) evoked firing in ChR2‐expressing LC neurons (Figure 2C), while yellow light stimulation (589 nm, 0.5 s, 2 mW) resulted in inhibition of NpHR‐expressing LC neurons (Figure 2E). Thus, transfection of ChR2 and NpHR allowed reversible optogenetic stimulation and inhibition of LC‐GABA neurons, respectively. We observed that optogenetic activation or inhibition of unilateral LC‐GABA neurons reversibly evoked significant increases and decreases in mechanical paw withdrawal threshold (PWT) and thermal paw withdrawal latency (PWL) on both sides in ChR2 and NpHR mice, respectively, relative to eYFP controls (Figure 2F–M). These data indicate that LC‐GABA neurons bidirectionally regulate mechanical and thermal pain thresholds on both sides.

We next employed the conditioned place preference or aversion (CPP or CPA) paradigm as showed in Figure 2N to examine whether repetitive stimulation or inhibition of LC‐GABA neurons leads to CPP or CPA. We observed that ChR2 but not eYFP mice developed CPP as ChR2 mice stayed longer in the blue‐light‐paired chamber (Figure 2O–Q). In contrast, optogenetic inhibition of LC‐GABA neurons in NpHR mice was sufficient to induce CPA (Figure 2R–T). Therefore, LC‐GABA neurons may be involved in reward and aversion processing.

Furthermore, to examine depression‐like behaviors, we employed three routine assays: the tail suspension test (TST), the forced swim test (FST), and the sucrose preference test (SPT). We observed that optogenetic stimulation of LC‐GABA neurons attenuated depression‐like behaviors: specifically, decreased immobility time in the TST and FST and improved sucrose preference in the SPT (Figure 2U–W). In contrast, inhibition of LC‐GABA neurons induced depression‐like behaviors: specifically, increased immobility time in the TST and FST and attenuated sucrose preference in the SPT (Figure 2U–W).

Collectively, these experiments demonstrate that LC‐GABA neurons bidirectionally modulate pain‐, reward‐, aversion‐ and depression‐like behaviors in mice.

### 2.3 Activation of Hyperactive LC‐GABA Neurons Mitigates Pain‐Associated Behaviors in Neuropathic Pain

**Figure 3:**
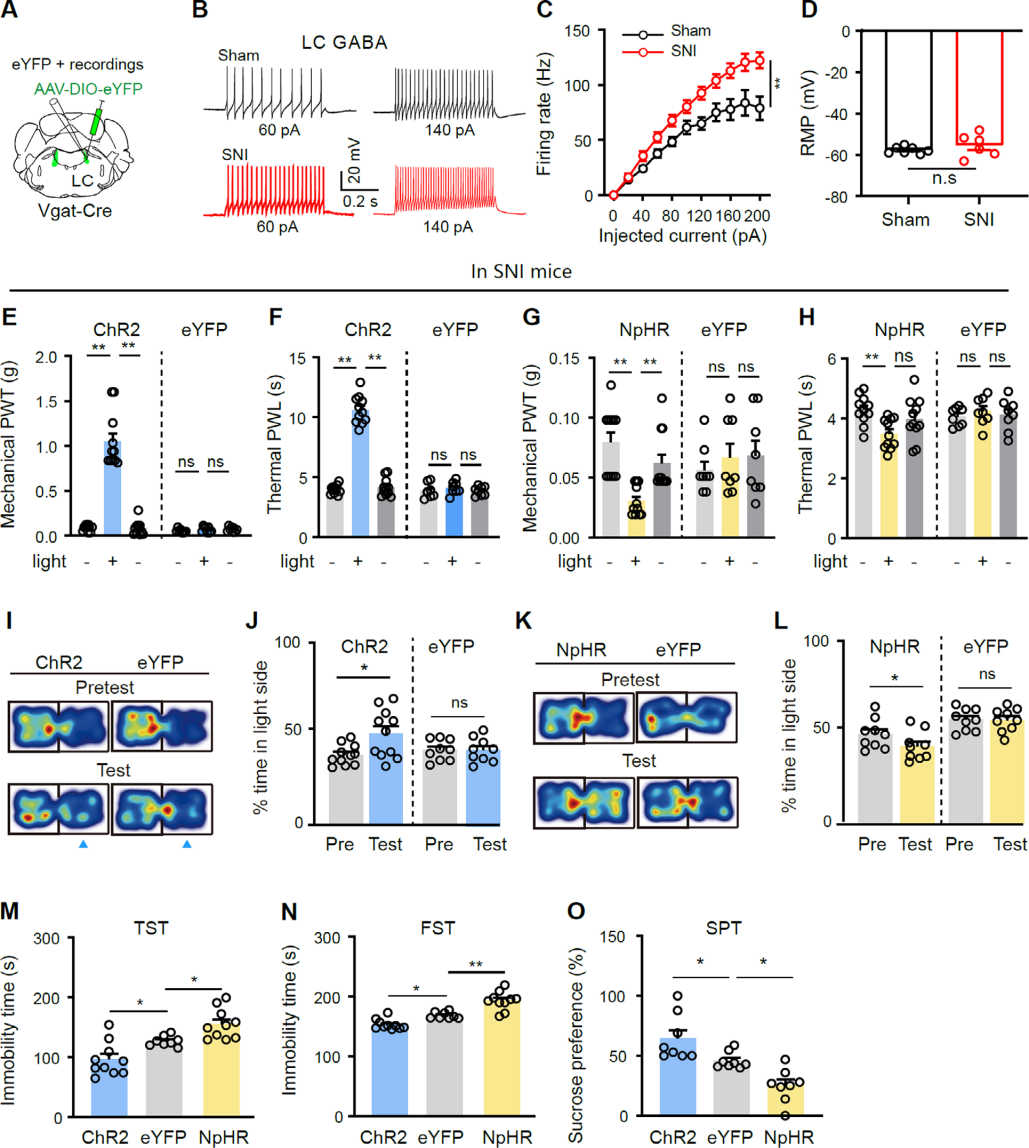
LC‐GABA neurons regulate pain thresholds and depression‐like behaviors in SNI mice. A) Whole‐cell patch‐clamp recording of eYFP‐labeled GABA neurons in the LC. B,C) Example traces and summary of firing in response to step current injection in LC‐GABA neurons from sham and SNI mice. *F*₍₁,₁₀₎ = 13.62, *P* = 0.004 for C); *n* = 5 in SNI, *n* = 7 in sham. D) Summary of RMP in LC‐GABA neurons from sham and SNI mice. *t *= 1.65, *P* = 0.32; *n* = 6 in SNI, *n* = 7 in sham. E–H) Mechanical paw withdrawal threshold (PWT) and thermal paw withdrawal latency (PWL) on the ipsilateral hind paw in ChR2, NpHR, and eYFP mice two weeks after SNI. Group: *F*₍₁,₁₇₎ = 134.8, *P* < 0.0001 for E); Group: *F*₍₁,₁₇₎ = 147.4, *P* < 0.0001 for F); *n* = 11 in ChR2, *n* = 8 in eYFP. Group: *F*₍₁,₁₇₎ = 0.86, *P* = 0.37; Interaction: *F*₍₂,₃₄₎ = 6.43, *P* = 0.003 for G). Group: *F*₍₁,₁₇₎ = 1.64, *P* = 0.22; Interaction: *F*₍₂,₃₄₎ = 5.5, *P* = 0.009 for H); *n* = 11 in NpHR, *n *= 8 in eYFP. I,J) Example heat maps (I) and quantification of percentage time spent in the blue‐light‐paired chamber during pretest and test sessions (J) in ChR2 and eYFP mice. Group: *F*₍₁,₁₇₎ = 17.02, *P *= 0.0007 for J); *n *= 11 in ChR2, *n* = 9 in eYFP. K,L) Example heat maps (K) and quantification of percentage time spent in the yellow‐light‐paired chamber during pretest and test sessions (L) in NpHR and eYFP mice. Group: *F*₍₁,₁₆₎ = 13.91, *P* = 0.002 for L); *n* = 9 in each group. M–O) Quantification of depression‐like behaviors in ChR2, NpHR, and eYFP mice five weeks after SNI. *F*₍₂,₂₅₎ = 16.07, *P* < 0.0001 for M); *F*₍₂,₂₅₎ = 32.87, *P* < 0.0001 for N); *F*₍₂,₂₁₎ = 15.06, *P* < 0.0001 for O). *n* = 10 in ChR2, *n* = 8 in eYFP, *n *= 10 in NpHR for M and N); *n* = 8 in each group for O). *P < 0.05. **P < 0.01; Two‐way repeated measures ANOVAs with Tukey's post‐hoc analysis for panels C, E–H, J, and L; One‐way ANOVAs with Tukey's post‐hoc analysis for panels M–O; Two‐tailed unpaired *t*‐test for panels D.

To examine the neuronal excitability in LC‐GABA neurons in neuropathic pain, we performed whole‐cell patch‐clamp recordings on LC‐GABA neurons labeled with AAV‐EF1α‐DIO‐eYFP in Vgat‐Cre mice 4 weeks after SNI surgery (**Figure** **3**A). SNI mice exhibited increased pain sensitivity since the first week after surgery and depression‐like behaviors characterized by increased immobility time in the TST and FST as well as reduced sucrose preference in the SPT 4–5 weeks after SNI surgery (Figure S1A–E, Supporting Information). We found that in SNI mice 4 weeks after surgery, LC‐GABA neurons exhibited enhanced frequencies in the firing evoked by depolarizing current injections but with unchanged resting membrane potentials (RMP), compared with sham mice (Figure 3B–D). These results suggest that LC‐GABA neurons are hyperactive in SNI mice comorbid with depression‐like behavior.

As optogenetic inhibition of LC‐GABA neurons is sufficient to induce hyperalgesia (Figure 2J–M), CPA (Figure 2R–T), and depression‐like behavior (Figure 2U–W) in naïve mice, mimicking the typical symptoms of neuropathic pain, we decided to investigate whether bidirectional modulation of LC‐GABA neurons alters pain hypersensitivity and depression‐like behavior in SNI mice. Considering that the LC is an important component in the descending pain pathway, we injected AAV‐EF1α‐DIO‐ChR2‐eYFP, AAV‐EF1α‐DIO‐NpHR‐eYFP or AAV‐EF1α‐DIO‐eYFP, and implanted an optical fiber into the LC of Vgat‐Cre mice on the same side as SNI surgery was performed. We assessed the effect of optogenetic stimulation and inhibition of LC‐GABA neurons on mechanical and thermal pain thresholds and ongoing pain‐associated place aversion 2 weeks after SNI surgery, and depression‐like behavior 5 weeks after SNI surgery. We observed that optogenetic activation of LC‐GABA neurons significantly increased mechanical PWT and thermal PWL in SNI mice (Figure 3E,F), while optogenetic inhibition of LC‐GABA neurons further decreased mechanical PWT and thermal PWL on the hind paw of the injured side in SNI mice (Figure 3G,H). Therefore, the hyperactivity of LC‐GABA neurons in SNI mice may provide a compensatory mechanism to limit hyperalgesia.

Furthermore, optogenetic activation of LC‐GABA neurons established CPP (Figure 3I,J), while inhibition of LC‐GABA neurons was sufficient to induce CPA (Figure 3K,L). Likewise, optogenetic activation of LC‐GABA neurons attenuated depression‐like behaviors (decreased immobility time in the TST and FST and enhanced sucrose preference in the SPT) in SNI mice (Figure 3M–O). In contrast, optogenetic inhibition of LC‐GABA neurons exacerbated depression‐like behaviors as demonstrated by increased immobility time in the TST, FST, and reduced sucrose preference in the SPT in SNI mice (Figure 3M–O). Therefore, stimulation of LC‐GABA neurons mitigated depressant‐like behaviors in SNI mice.

Because norepinephrine is a major neurotransmitter of the LC and is known to induce analgesia in the spinal cord.^[^ ^6^ ^]^ We hypothesized that LC‐GABA neurons‐induced antinociception might be attenuated by spinal cord delivery of α2 adrenergic receptor antagonist. To test this, we specifically transfected hM3Dq into unilateral LC‐GABA neurons in mice (Figure S2A,B, Supporting Information). We measured mechanical PWT and thermal PWL before and after intraperitoneal administration of Clozapine‐N‐oxide (CNO) to activate LC‐GABA neurons or after administration of CNO combined with intrathecal delivery of an α2‐adrenergic‐receptor antagonist yohimbine (Figure S2C, Supporting Information). We observed that yohimbine blocked the analgesic effects of chemogenetic activation of LC‐GABA neurons in both naïve and SNI mice (Figure S2D–G, Supporting Information).

Altogether, these data suggest that spinal norepinephrine may play a role in the descending pathway of LC‐GABA neurons and that stimulating LC‐GABA neurons could have therapeutic potential to treat sensory and affective components of neuropathic pain.

### 2.4 LC‐GABA Neurons Modulate Pain‐Like Response in LC‐NA Neurons

**Figure 4:**
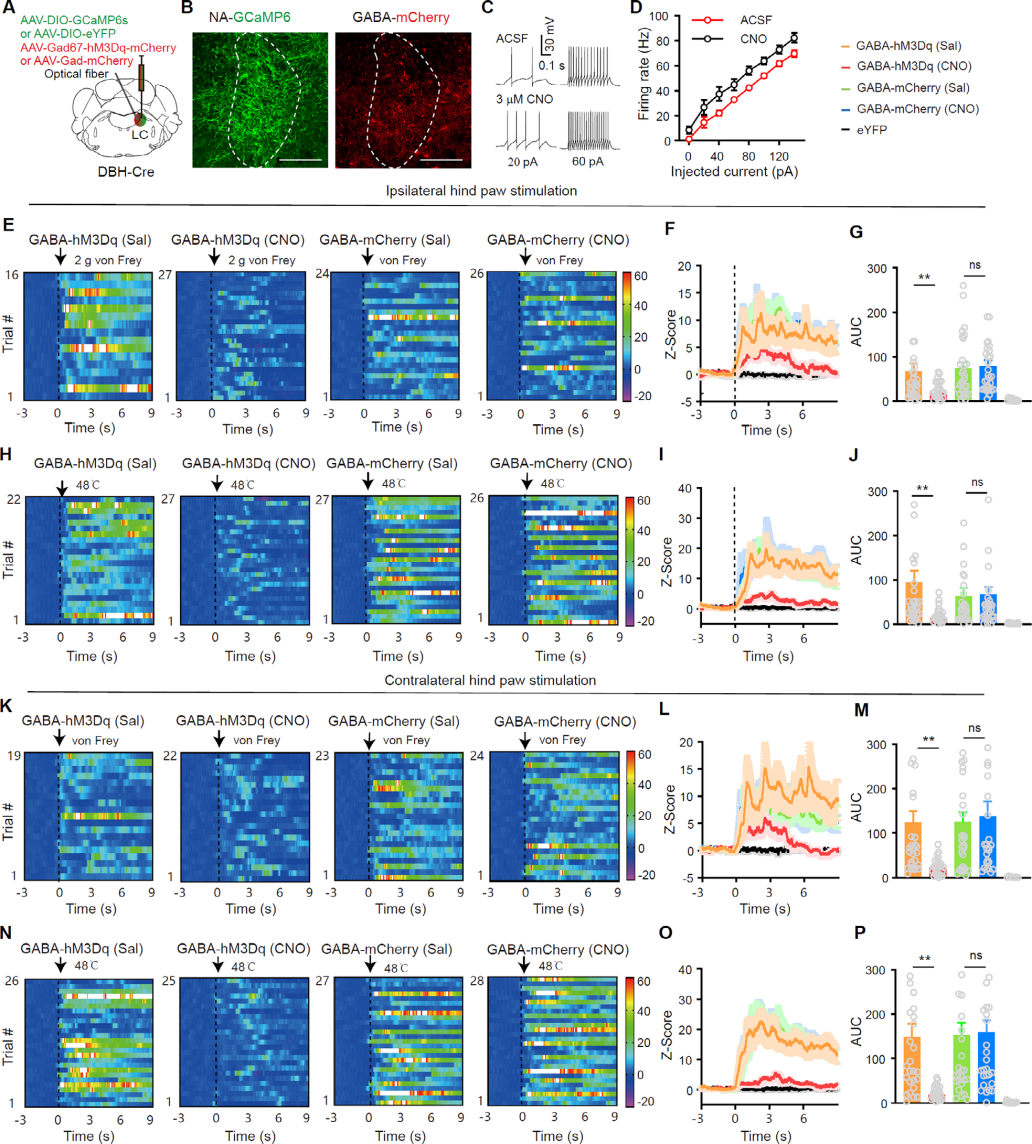
Chemogenetic inhibition of LC‐GABA neurons attenuates the responses of NA neurons to pain‐like stimuli. A) Schematic diagram for virus injection and optical fiber placement in fiber photometry experiments. B) GCaMP6 and hM3Dq expression in NA neurons and GABA neurons in the LC, respectively. C,D) Example traces and summary of current injection evoked action potentials in LC‐GABA neurons during artificial cerebrospinal fluid (ACSF) and Clozapine‐N‐Oxide (CNO, 3 µm) perfusion. *F*_(1, 4)_ = 18.95, *P* = 0.012, *n* = 5 cells in each group for D). E–J) Heat maps (E and H), average traces (F and I), and summary (G and J) of GCaMP6 signal in LC‐NA neurons of mice receiving von Frey filament (2 g) or thermal stimulation on ipsilateral hind paws with saline or CNO administration. hM3Dq (saline vs CNO), *t *= 2.57, *P* = 0.013 for G); mCherry (saline vs CNO), *t *= 0.22, *P* = 0.83 for G); hM3Dq (saline versus CNO), *t* = 4.02, *P* = 0.0002 for J); mCherry (saline vs CNO), *t* = 0.35, *P* = 0.73 for J); *n* = 30 trials from 5 mice in each group. K–P) Heat maps (K and N), average traces (L and O), and summary (M and P) of GCaMP6s signal in LC‐NA neurons of mice receiving von Frey filament (2 g) or thermal stimulation on contralateral hind paws with saline or CNO administration. hM3Dq (saline vs CNO), *t* = 2.91, *P* = 0.005 for M); mCherry (saline vs CNO), *t* = 0.12, *P* = 0.90 for M); hM3Dq (saline vs CNO), *t* = 4.29, *P* < 0.0001 for P); mCherry (saline vs CNO), *t *= 0.16, *P* = 0.87 for P); *n* = 30 trials from 5 mice in each group. Dashed lines indicate stimulus onset. ** *P *< 0.01; Two‐way repeated measures ANOVAs in panel D. Two‐tailed unpaired *t*‐test in panels G, J, M, P. Scale bars: 100 µm.

Since LC‐GABA neurons send synaptic inputs to NA neurons within the LC,^[^ ^5a,b^ ^]^ which are known to play important roles in chronic pain‐associated behaviors,^[^ ^3^, ^4^ ^]^ we investigated whether LC‐GABA neurons attenuate pain‐like response in LC‐NA neurons. To test this, we injected AAV‐EF1α‐DIO‐GCaMP6s or AAV‐ EF1α‐DIO‐eYFP into the unilateral LC of DBH‐Cre mice to record GCaMP6 signals in NA neurons. Additionally, we injected AAV‐GAD67‐hM3Dq‐mCherry or AAV‐GAD67‐mCherry into the LC to transfect hM3Dq or mCherry into LC‐GABA neurons on the same side (**Figure** **4**A,B; Figure S3A,B, Supporting Information).

We first recorded the GCaMP6 signal of LC‐NA neurons in both naïve and SNI mice (Figure S4, Supporting Information). LC‐NA neurons showed significant responses to suprathreshold mechanical (2 g von Frey) and thermal (48 °C) stimulation, but not to subthreshold mechanical stimulation in either naïve (0.4 g) or SNI (0.008 g) mice (Figure 4E–P; Figure S4B,C, Supporting Information). In contrast, such responses were absent in eYFP control mice (Figure 4E–P; Figure S4B,C, Supporting Information). Additionally, LC‐NA neurons responded significantly to aversive (air puff) and rewarding (sucrose licking) stimuli, with enhanced aversive responses in SNI mice, while responses to rewarding stimuli remained comparable between naïve and SNI mice (Figure 4D,E). To explore the role of LC‐GABA neurons, we examined responses of LC‐NA neurons to suprathreshold mechanical (2 g von Frey) and thermal (48 °C) stimulation following intraperitoneal administration of CNO (3 mg k^−1 ^g) to activate LC‐GABA neurons in naïve mice. CNO enhanced LC‐GABA neuron activity (Figure 4C,D), which significantly attenuated the GCaMP6 signals of LC‐NA neurons evoked by von Frey filament or thermal stimulation on either hind paw (Figure 4E–P). In control mice with mCherry virally transfected into LC‐GABA neurons, CNO did not alter the responses of LC‐NA neurons to mechanical and thermal stimulation (Figure 4E–P). Together, these results demonstrate that LC‐NA neurons are activated by mechanical and thermal stimuli, and activation of LC‐GABA neurons strongly inhibits this activation, limiting responses of LC‐NA neurons to mechanical and thermal stimulation.

### 2.5 Chemogenetic Activation of LC‐GABA Neurons Regulates Pain‐Associated Behaviors with or without Activation of LC‐NA Neurons

**Figure 5:**
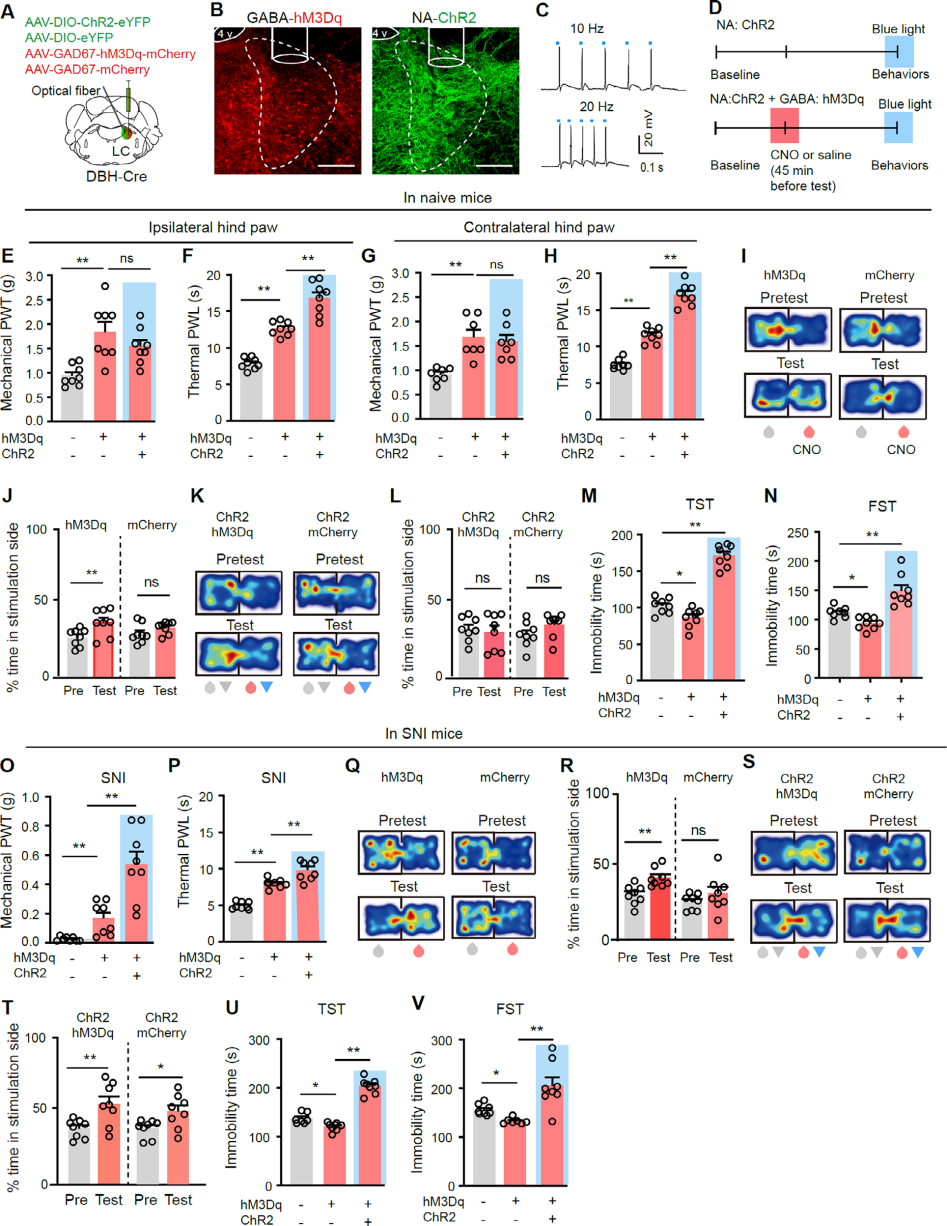
Activation of LC‐NA neurons variably affects the modulation of pain thresholds and depression‐like behaviors by stimulation of LC‐GABA neurons in naïve and SNI mice. A) Schematic diagram for virus injection and optical fiber placement for selective activation of GABA neurons and NA neurons in the LC, respectively. B) Example images of expression of hM3Dq and ChR2 in the LC. C) Example traces of action potentials induced by blue light in a ChR2‐eYFP‐labeled NA neuron. D) Schematic diagram of experimental design. E–H) Mechanical PWT and thermal PWL on hind paws of three different groups. Ipsilateral PWT, *F*_(2, 14)_ = 8.98, *P* = 0.003 for E); Ipsilateral PWL, *F*_(2, 14)_ = 109.4, *P* < 0.0001 for F); Contralateral PWT, *F*_(2, 12)_ = 10.57, *P* = 0.002 for G); Contralateral PWL, *F*_(2, 14)_ = 125.1, *P* < 0.0001 for H); *n* = 8 in each group. I,J) Example heat maps (I) and quantification of % time spent in CNO paired chamber during pretest and test sessions (J) in hM3Dq and mCherry mice. Group: *F*_(1, 14)_ = 10.23, *P* = 0.006 for J); *n* = 8 in each group. K,L) Example heat maps (K) and quantification of % time spent in CNO plus blue light paired chamber during pretest and test sessions (L) in two groups of mice. Group: *F*_(1, 14)_ = 0.035, *P* = 0.85 for L); *n* = 8 in each group. M,N) Immobility time in TST and FST. *F*_(1, 21)_ = 95.68, *P* < 0.0001 for M); *F*_(1, 21)_ = 20.78, *P* < 0.0001 for N); *n *= 8 in each group. O,P) Mechanical PWT and thermal PWL on SNI‐injured hind paws of three groups of mice. SNI was performed on the ipsilateral side to virus injection. PWT, *F*_(2, 14)_ = 29.39, *P* < 0.0001 for O); PWL, *F*_(2, 14)_ = 63.24, *P* < 0.0001 for P); *n *= 8 in each group. Q,R) Example heat maps (Q) and quantification of % time spent in CNO paired chamber during pretest (Pre) and test sessions (R) in hM3Dq and mCherry mice. Group: *F*_(1, 14)_ = 5.09, *P* = 0.041 for R); *n *= 8 in each group. S,T) Example heat maps (S) and quantification of % time spent in CNO plus blue light‐paired chamber during pretest and test sessions (T) in two groups of mice. Pre versus Test: *F*_(1, 14)_ = 20.94, *P* = 0.0004 for T); *n* = 8 in each group. U) Immobility time in the TST, *F*_(2, 20)_ = 107.4, *P* < 0.0001; V) Immobility time in the FST, *F*_(2, 20)_ = 12.81, *P *= 0.0003; *n* = 7–8 in each group. *P < 0.05. **P < 0.01; One‐way ANOVAs with Tukey's post‐hoc analysis for panels E–H, M–P, U, and V; Two‐way repeated measures ANOVAs with Tukey's post‐hoc analysis for panels J, L, R and T. Scale bars: 100 µm.

Data in Figure 4 suggest that LC‐GABA neurons, when activated, may inhibit LC‐NA neurons. To examine whether this inhibition contributes to the modulation of pain‐associated behaviors by LC‐GABA neuron activation, we designed a strategy to simultaneously activate LC‐NA and LC‐GABA neurons to address whether the effects of LC‐GABA neuron activation can be counteracted. Specifically, we injected AAV‐EF1α‐DIO‐ChR2‐eYFP or AAV‐EF1α‐DIO‐eYFP, along with AAV‐GAD67‐hM3Dq‐mCherry or AAV‐GAD67‐mCherry into the LC and implanted optical fiber into the LC of DBH‐Cre mice, to achieve optogenetic activation of LC‐NA neurons and chemogenetic activation of LC‐GABA neurons, respectively (**Figure** **5**A,B,D; Figure S5A,B, Supporting Information). Optogenetic stimulation could drive LC‐NA neurons to fire up to 20 Hz (Figure 5C).

We first tested whether LC‐NA neurons modulate pain‐ and depression‐like behaviors under our experimental condition. Unilateral optogenetic activation of LC‐NA neurons reversibly increased PWT and PWL on both hind paws (Figure S5C–J, Supporting Information) and produced anti‐depressant‐like behaviors in the TST and FST (decreased immobility time) (Figure S5K,L, Supporting Information), but did not establish either CPP or CPA (Figure S5M,N, Supporting Information) in naïve mice. We then investigated the effects of LC‐NA neuron activation in SNI mice. SNI surgery was performed ipsilateral to virus injection, and pain thresholds on the hind paw on the same side were tested. Unilateral optogenetic activation of LC‐NA neurons increased PWT and PWL on the hind paw on the injured side (Figure S5O–R, Supporting Information), consistent with the observation in naïve mice (Figure S5C–J, Supporting Information). However, activation of LC‐NA neurons induced CPP (Figure S5S,T, Supporting Information) and exacerbated depression‐like behaviors in the TST and FST in SNI mice (Figure S5U,V, Supporting Information). None of these behaviors were affected by blue light illumination of the LC in eYFP mice (Figure S5P,R,T, Supporting Information).

We next examined whether LC‐NA neurons influence the effect of LC‐GABA neurons on pain‐related behaviors. Acute unilateral chemogenetic activation of LC‐GABA neurons produced effects similar to those observed with optogenetic activation of LC‐GABA neurons (Figure 2), including increased PWT and PWL on both hind paws (Figure 5E–H), CPP (Figure 5I,J), and antidepressant‐like effects in the TST and FST (decreased immobility time) (Figure 5M,N) in naïve mice. Optogenetic activation of LC‐NA neurons during activation of LC‐GABA neurons increased PWT and PWL on both hind paws (Figure 5E–H), did not induce CPP or CPA (Figure 5K,L), but led to depression‐like behaviors in naïve mice (Figure 5M,N). In SNI mice, unilateral chemogenetic activation of LC‐GABA neurons increased PWT and PWL on the hind paw on the injured side, and this effect was enhanced by simultaneous optogenetic activation of LC‐NA neurons (Figure 5O,P). Chemogenetic activation of LC‐GABA neurons alone or together with optogenetic activation of LC‐NA neurons established CPP (Figure 5Q–T). While chemogenetic activation of LC‐GABA neurons alone mitigated depression‐like behavior in SNI mice, simultaneous activation of LC‐GABA and LC‐NA neurons exacerbated these behaviors (Figure 5U,V).

Together, these data suggest that in physiological conditions, activation of LC‐NA neurons may counteract the activation of LC‐GABA neurons‐induced CPP and anti‐depressant effect, without attenuating the elevation of pain threshold; in neuropathic pain, activation of LC‐NA neurons variably altered behavioral effects of LC‐GABA neuron activation: enhanced analgesic effect, maintaining CPP establishment, but counteracting anti‐depressant effects. These findings highlight the complex interplay between LC‐NA and LC‐GABA neurons in modulating pain‐ and affect‐related behaviors under different conditions.

### 2.6 Regulation of Pain‐Associated Behaviors by LC‐GABA Neurons Do Not Depend on LC‐NA Neurons

**Figure 6:**
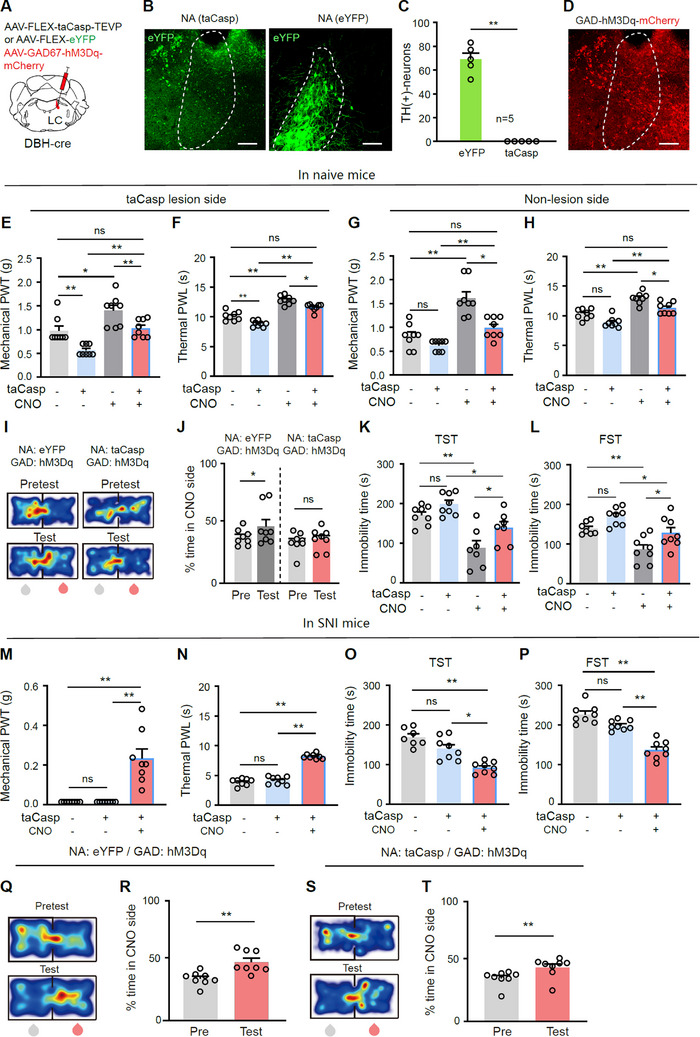
Ablation of LC‐NA neurons does not affect the antinociceptive and antidepressant effects of LC‐GABA neuron activation. A) Schematic diagram for virus injection for NA neuron ablation and chemogenetic activation of LC‐GABA neurons. B,C) Example images and summary of ablation of LC‐NA neurons with taCasp3. *t *= 8.89, *P* < 0.0001, *n *= 5 in each group. D) Example image of hM3Dq expression in LC‐GABA neurons. E–H) Mechanical PWT and thermal PWL in four different groups. Ipsilateral PWT, *F*_(3, 21)_ = 17.94, *P* < 0.0001for E); Ipsilateral PWL, *F*_(3, 21)_ = 27.27, *P* < 0.0001 for F); Contralateral PWT, *F*_(3, 21)_ = 49.89, *P* < 0.0001 for G); Contralateral PWL, *F*_(3, 21)_ = 24.08, *P* < 0.0001 for H); *n *= 8 in each group. I,J) Example heat maps (I) and quantification of % time spent in CNO paired chamber during pretest and test sessions (J) in two groups of mice. Pre versus Test: *F*_1, 14)_ = 5.58, *P* = 0.033 for J); *n* = 8 in each group. K) Immobility time in the TST, *F*_(3, 21)_ = 12.32, *P* = 0.0031. L) Immobility time in the FST, *F*_(3, 21)_ = 27.02, *P* < 0.0001; *n* = 8 in each group. M,N) Mechanical PWT and thermal PWL in three different groups of SNI mice. *F*_(2, 21)_ = 21.92, *P* < 0.0001 for M); *F*_(2, 21)_ = 150.6, *P* < 0.0001 for N); *n* = 8 in each group. O,P) Immobility time in three different group mice in the TST and FST. TST, *F*_(2, 21)_ = 22.26, *P *< 0.0001 for O); FST, *F*_(2, 21)_ = 40.44, *P *< 0.0001 for P); *n *= 8 in each group. Q–T) Example heat maps (Q and S) and quantification of % time spent in CNO‐paired chamber during pretest and test sessions (R and T) in two groups of mice. *t* = 3.69, *P* = 0.008 for R); *t *= 5.89, *P* = 0.0006 for T); *n *= 8 in each group. *P < 0.05. **P < 0.01; One‐way ANOVAs with Tukey's post‐hoc analysis for panels E–H, K–P; Two‐way repeated measures ANOVAs with Tukey's post‐hoc analysis for panel J; Two‐tailed paired *t*‐test for panels R and T. Scale bars: 100 µm.

Since activation of LC‐NA neurons heterogeneously affects the behavioral outcomes of LC‐GABA neuron activation in naïve and SNI mice, we examined how ablation of LC‐NA neurons affects these outcomes. To achieve this, we transfected hM3Dq‐mCherry into LC‐GABA neurons and taCasp3 or eYFP into LC‐NA neurons in DBH‐Cre mice to combine chemogenetic modulation of LC‐GABA neurons with genetic ablation of LC‐NA neurons (**Figure** **6**A–D). Successful ablation of LC‐NA neurons was confirmed 3 weeks after the injection of AAV‐FLEX‐taCasp‐TEVP into the LC by a dramatic reduction of TH‐immunoreactive neurons in the LC (Figure 6B,C).

We first tested the pain threshold of naïve mice. On the ipsilateral hind paw, ablation of LC‐NA neurons reduced both mechanical PWT and thermal PWL, compared to mice with intact LC‐NA neurons (Figure 6E,F). CNO activation of LC‐GABA neurons increased PWT and PWL in both intact and ablation groups, although the analgesic effect was less pronounced in mice with ablation of LC‐NA neurons (Figure 6E,F). On the contralateral hind paw (Figure 6G,H), PWT and PWL did not differ between intact and ablation groups, but CNO activation of LC‐GABA neurons again produced a weaker analgesic effect in mice with ablation of LC‐NA neurons (Figure 6G,H). These data indicate that the analgesic effect of LC‐GABA neurons does not completely depend on LC‐NA neurons.

Next, we examined reward‐ and depression‐like behaviors. Chemogenetic activation of LC‐GABA neurons established CPP in mice with intact NA neurons, but this effect was absent in mice with ablation of NA neurons (Figure 6I,J). In contrast, ablation of LC‐NA neurons did not affect depression‐like behaviors as showed in the TST and FST (Figure 6K,L), and CNO activation of LC‐GABA neurons alleviated depression‐like behaviors in both groups (Figure 6K,L). These findings indicate that LC‐NA neurons may be necessary for LC‐GABA neurons to modulate reward processing but not depression‐like behavior in naïve mice.

We next tested pain and depression‐like behaviors in SNI mice. Ablation of LC‐NA neurons did not alter PWT, PWL, or immobility time in the TST and FST (Figure 6M–P). CNO activation of LC‐GABA neurons substantially elevated PWT and PWL, reduced immobility time, and established CPP in mice with and without LC NA neuron ablation (Figure 6M–T).

Furthermore, to avoid the potential compensatory effects of permanent ablation, we conducted the experiments using optogenetic inhibition. In DBH‐Cre mice, AAV‐DIO‐GAD67‐hM3Dq‐mCherry and AAV‐DIO‐NpHR‐eYFP were injected into the LC to transfect GABA neurons and NA neurons, respectively (Figure S6A,B, Supporting Information). Similar to the ablation of LC‐NA neurons, inhibition of LC‐NA neurons in naïve mice reduced mechanical PWT and thermal PWL on the ipsilateral hind paw and induced depression‐like behavior (Figure S6C–F,I,J, Supporting Information). In mice whose LC‐NA neurons were inhibited with CNO, optogenetic activation of LC‐GABA neurons still produced an analgesic effect, alleviated depression‐like behaviors in LC‐NA‐inhibited mice, but failed to induce CPP (Figure S6C–J, Supporting Information). In SNI mice, optogenetic inhibition of LC‐NA neurons did not affect pain thresholds or depression‐like behaviors, and optogenetic activation of LC‐GABA neurons increased PWT and PWL, reduced immobility time in TST and FST, and induced CPP when LC‐NA neurons were inhibited (Figure S6K–P, Supporting Information).

Altogether, these results suggest that the analgesic and antidepressant effects of LC‐GABA neurons do not fully depend on LC‐NA neurons, while their reward effects in naïve mice require LC‐NA neurons remain activity at certain levels.

We further investigated the potential roles of spinal norepinephrine and serotonin in pain modulation of LC‐GABA neurons (Figure S7A–C, Supporting Information). As showed in Figure S7D–G (Supporting Information), blockade of either α2‐adrenergic receptors or 5‐HT3 or 5HT1/2 receptors by intrathecally delivering antagonists (yohimbine, ondansetron, or metergoline) significantly compromised the analgesic effects of chemogenetic activation of LC‐GABA neurons in mice with and without ablation of LC‐NA neurons. These data suggest that spinal serotoninergic and noradrenergic receptors are involved in the pain modulation of LC‐GABA neurons. Note that yohimbine blocked the antinociceptive effect of LC‐GABA neuron activation in mice with LC‐NA neuron ablation. It seems inconsistent with our observation that LC‐GABA and LC‐NA neurons regulate pain through different pathways. It has been known that in addition to the LC (A6 area), the spinal cord receives NA inputs from the A5 and A7 areas in the ventral and dorsolateral pons^[^ ^7^ ^]^ and these NA neurons are connected with a number of other nuclei influencing pain‐related behavior and receive projections from the PAG.^[^ ^7a,c^ ^]^ Therefore, the ablation of LC‐NA neurons alone may not be sufficient to completely disrupt the descending pain pathway activated by LC‐GABA neurons.

### 2.7 LC‐GABA Neurons Innervate GABA and Glutamatergic Neurons in the PAG Differently

To determine which downstream nuclei participate in sensory and emotional modulation by LC‐GABA neurons, we next traced the axonal projections of LC‐GABA neurons by injecting AAV‐hSyn‐DIO‐mGFP‐synaptophysin‐mRuby into the LC of Vgat‐Cre mice (Figure S8A, Supporting Information). This viral vector labels LC‐GABA neurons and their processes with mGFP and axonal terminals with mRuby (Figure S8B, Supporting Information). mRuby‐labeled structures were distributed in a large collection of cortical and subcortical areas (Figures S8C,S10, Supporting Information). Notably, there was a dense signal of mRuby in the PAG. As a complementary approach to determine the downstream nuclei of LC‐GABA neurons, AAV‐EF1α‐DIO‐ChR2‐eYFP was injected into the LC of Vgat‐Cre mice to label LC‐GABA neurons with ChR2‐eYFP (Figure S9A,B, Supporting Information). We analyzed the density of eYFP‐labeled axons from LC‐GABA neurons (Figure S9C, Supporting Information). Similar to mRuby‐labeled terminals, the axonal projections of LC‐GABA neurons were broadly distributed in the brain, including the vlPAG (Figures S8C,S9C,S10, Supporting Information). Given the role of the vlPAG in pain descending inhibition system,^[^ ^8^ ^]^ we hypothesized that LC‐GABA neurons may exert analgesic effect by suppressing vlPAG neurons.

**Figure 7:**
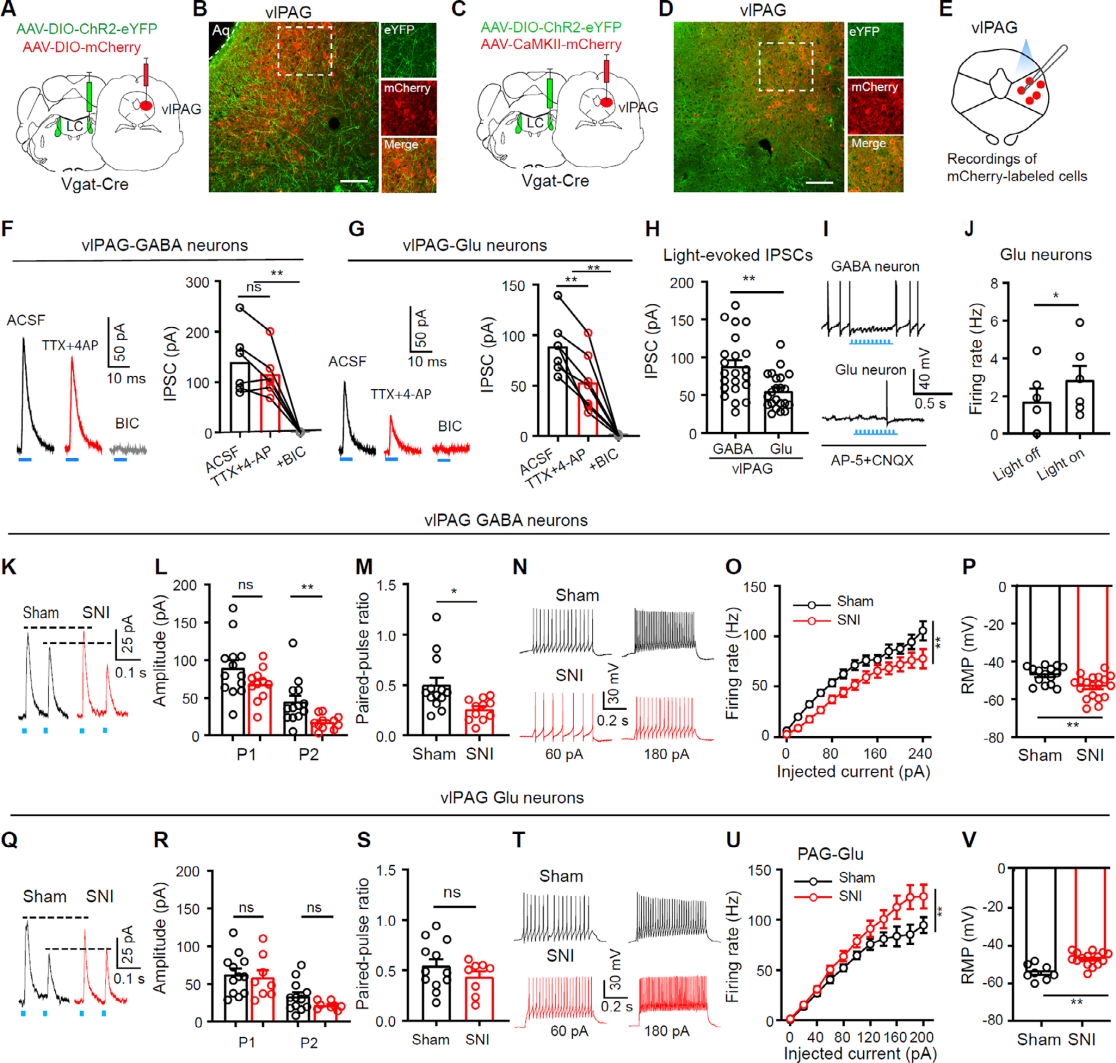
Modulation of LC‐GABA projection in the vlPAG in SNI mice. A–D) Schematic diagrams for virus injection (A and C) and example images of virus expression in the ventrolateral Periqueductal Gray (vlPAG) (B and D). E) Schematic diagram of patch‐clamp recordings in mCherry‐labeled GABA or Glu neurons in the vlPAG. F) Example traces and quantification of blue light (5 ms, 1 mW) evoked‐inhibitory postsynaptic currents (IPSCs) recorded at −40 mV from vlPAG‐GABA neurons in ACSF, TTX (1 µm) with 4‐AP (100 µm), or bicuculline (BIC, 10 µm); *F*_(2, 10)_ = 27.97, *P* < 0.0001, *n *= 6 cells. G) Example traces and quantification of blue light (5 ms, 1 mW) evoked‐IPSCs recorded at −40 mV from vlPAG‐Glu neurons in ACSF, Tetrodotoxin (TTX, 1 µm) with 4‐Aminopyridine (4‐AP, 100 µm), or bicuculline (BIC, 10 µm); *F*_(2, 12)_ = 44.12, *P* < 0.0001, *n* = 7 cells. H) Summary of the amplitude of blue light‐evoked IPSCs in GABA neurons and glutamatergic (Glu) neurons in the vlPAG; *t* = 3.30, *P* = 0.002, *n* = 22 cells from 4 mice in each group. I) Example traces of light‐evoked changes in firing during bath perfusion with AP‐5 (30 µm) and CNOX (10 µm). J) Quantitation of action potential firing rate before and during blue light; *t *= 3.43, *P* = 0.018, *n* = 6 cells from 5 mice. K and L) Example traces (K) and summary data (L) of blue light (5 ms pulses, 20 Hz) evoked‐IPSCs recorded at −40 mV from mCherry‐labeled vlPAG‐GABA neurons in sham and SNI mice. L) The amplitude of the first and second IPSCs (P1 and P2) on mCherry‐labeled vlPAG‐GABA neurons in sham and SNI mice. *P*1, *t* = 1.64, *P* = 0.12; *P*2, *t* = 2.91, *p* = 0.0081; unpaired *t*‐test. *n* = 13 cells in sham, *n *= 11 cells in SNI. M) Paired‐pulse ratio, *t *= 2.72, *P* = 0.012, *n* = 13 cells from 4 sham mice, *n* = 11 cells from 4 SNI mice. N and O) Example action potential responses to step current injection (N) and summary data of firing rate (O) from mCherry‐labeled vlPAG‐GABA neurons in sham and SNI mice. Sham versus SNI: *F*_(1, 31)_ = 12.48, *P* = 0.001 for O); *n* = 18 cells from 4 sham mice, *n* = 15 cells from 4 SNI mice. P) Summary data of the RMP of mCherry‐labeled vlPAG‐GABA neurons in sham and SNI mice; *t *= 3.3, *P* = 0.0024; *n* = 14 cells from 4 sham mice, *n* = 19 cells from 4 SNI mice. Q and R) Example traces (Q) and summary (R) of blue light (5 ms pulses, 20 Hz) evoked‐IPSCs recorded at −40 mV from mCherry‐labeled vlPAG‐Glu neurons in sham and SNI mice. R) The amplitude of the first and second IPSCs on mCherry‐labeled vlPAG‐Glu neurons in sham and SNI mice; *P*1, *t* = 0.33, *P* = 0.74; *P*2, *t *= 1.64, *p* = 0.12; unpaired *t*‐test; *n* = 12 cells in sham, *n* = 8 cells in SNI. S) Paired‐pulse ratio, *t *= 1.14, *P* = 0.27; *n* = 12 cells from 4 sham mice, *n *= 8 cells from 4 SNI mice. T,U) Example responses of firing to step current injection (T) and summary data of firing rate (U) in mCherry‐labeled vlPAG‐Glu neurons of sham and SNI mice; Sham versus SNI: *F*_(1, 20)_ = 7.97, *P* = 0.010 for U); *n* = 10 cells from 4 sham mice, *n* = 12 cells from 4 SNI mice. V) Summary of the resting membrane potentials (RMP) from mCherry‐labeled vlPAG‐Glu neurons in sham and SNI mice; *t* = 4.49, *P* = 0.0002, *n* = 8 cells from 4 sham mice, *n* = 12 cells from 4 SNI mice. *P < 0.05. **P < 0.01; Two‐tailed unpaired *t*‐test for panels H, J, L, M, P, R, S, and V; One‐way repeated measures ANOVAs with Tukey's post‐hoc analysis for panels F and G; Two‐way repeated measures ANOVAs with Tukey's post‐hoc analysis for panels O and U. Scale bars: 100 µm.

Glutamatergic (Glu) and GABA neurons are two major neuronal types in the vlPAG.^[^ ^8d^ ^]^ To characterize the LC^GABA^‐vlPAG projection, we combined the optogenetics with patch‐clamp recording (**Figure** **7**A–E). AAV‐EF1α‐DIO‐ChR2‐eYFP was injected into the LC of Vgat‐Cre mice to enable optogenetic stimulation of LC‐GABA neurons and their axons (Figure 7A,C). To label vlPAG GABA or Glu neurons, Vgat‐Cre mice received AAV‐EF1α‐DIO‐ChR2‐eYFP injection into the LC were divided into two groups, which received injection of AAV‐EF1α‐DIO‐mCherry or AAV‐CaMKII‐mCherry into the vlPAG, respectively (Figure 7A,C). Dense eYFP‐positive fibers from LC‐GABA neurons were observed surrounding mCherry‐positive GABA or Glu neurons in the vlPAG (Figure 7B,D). Patch‐clamp recording from either vlPAG GABA or Glu neurons showed that blue light elicited inhibitory postsynaptic currents (eIPSCs) at a holding voltage of −40 mV in both neurons. The light‐evoked eIPSCs were resistant to tetrodotoxin (TTX, 0.5 µm) along with 4‐aminopyridine (4‐AP, 100 µm), but were abolished by bicuculline (BIC, 10 µm) (Figure 7F,G). These data indicate that LC‐GABA neurons send monosynaptic innervation to both vlPAG GABA Glu neurons. Interestingly, the amplitude of eIPSCs in vlPAG‐GABA neurons was significantly greater than that in vlPAG‐Glu neurons. As shown in Figure 7I,J, blue light reliably inhibited firing in vlPAG‐GABA neurons (20/20 cells), whereas increased firing rate in vlPAG‐Glu neurons (Figure 7I,J). These electrophysiological data suggest that LC‐GABA neurons may recruit a disinhibitory circuit to enhance the activity of vlPAG‐Glu neurons, probably through vlPAG‐GABA neurons.

### 2.8 The Projection from LC‐GABA Neurons to vlPAG GABA and Glu Neurons are Differentially Modified in Neuropathic Pain Mice

We next investigated whether the LC^GABA^‐vlPAG projection is modified in neuropathic pain. To address this, we applied the viral vector‐assisted labeling approach depicted in Figure 7A,C. Subsequently, we performed SNI surgery ipsilateral to the side that the viral vector was injected. 4 weeks later, we performed whole‐cell patch‐clamp recording on mCherry‐labeled GABA and Glu neurons in the vlPAG. Blue light pulses (5 ms, 2 mW, 50 ms interval) reduced the amplitude of the second IPSC (P2) but had no significant effect on the amplitude of the first IPSC (P1) (Figure 7K,L). This resulted in a significant reduction of the paired‐pulse ratio (PPR) in vlPAG‐GABA neurons of SNI mice compared to sham mice (Figure 7K,M). Furthermore, GABA neurons responsive to blue light exhibited decreased firing rates and more hyperpolarized resting membrane potentials in SNI mice, compared to sham mice (Figure 7N–P). On the contrary, vlPAG‐Glu neurons displayed no change in the amplitude of the first and second IPSC and PPR of blue light‐evoked IPSCs (Figure 7Q–S), but increased firing rates and more depolarized membrane potentials (Figure 7T–V) in SNI mice, compared to sham mice. These data suggest that SNI preferentially enhances the LC^GABA^–vlPAG^GABA^ projection; in the vlPAG, GABA neurons transit toward a more inhibited state whereas Glu neurons become more active in SNI mice. The altered excitability in vlPAG GABA and Glu neurons in SNI mice may be consistent with the changes in the strength of inhibitory synaptic inputs they received in the vlPAG circuit: an increase in spontaneous IPSC frequency in vlPAG‐GABA neurons, but a reduction in spontaneous IPSC frequency in vlPAG‐Glu neurons (Figure S11A–F, Supporting Information). However, the link between the alterations in synaptic inputs and excitability of vlPAG GABA and Glu neurons warrants further investigations with neuromodulation approaches.

### 2.9 Activation of the LC^GABA^‐vlPAG Projection Alleviates Pain‐ and Depression‐Like Behaviors

**Figure 8:**
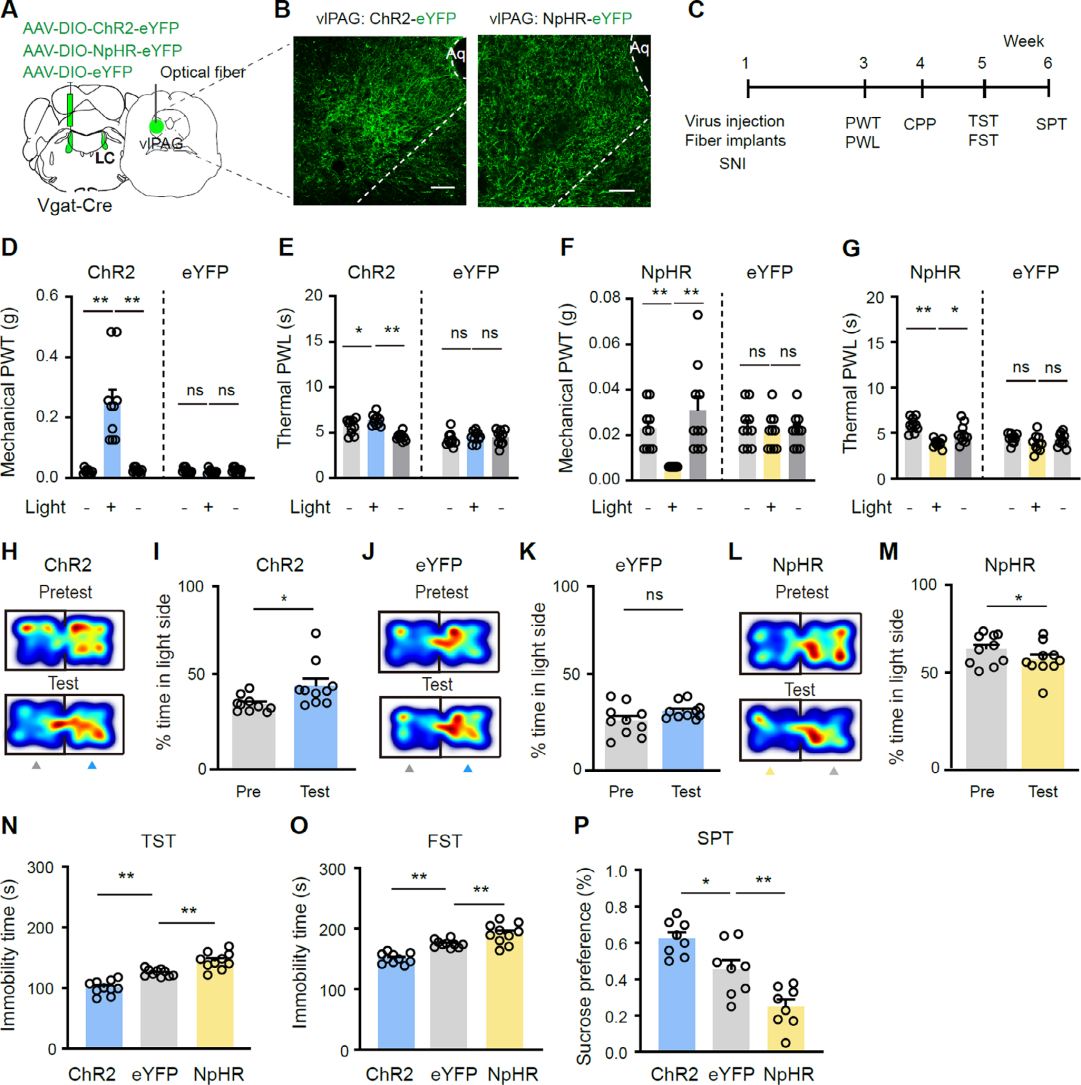
The LC‐vlPAG GABAergic projection modulates pain thresholds and depression‐like behaviors in SNI mice. A) Virus injection and optical fiber placement for selective activation or inhibition of the LC^GABA^‐vlPAG projection. B) Example images of ChR2‐ and NpHR‐labeled axonal terminals of LC‐GABA neurons within the vlPAG. C) Schematic diagram of experimental design. D,E) The effect of blue light illumination on mechanical PWT and thermal PWL on the ipsilateral hind paw in ChR2‐ and eYFP‐ expressing mice 2 weeks after SNI; Group: *F*_(1, 18)_ = 26.96, *P* < 0.0001 for D); Group: *F*_(1, 18)_ = 23.03, *P* = 0.0001 for E); *n* = 10 in each group. F,G) The effect of yellow light illumination on mechanical PWT and thermal PWL on the ipsilateral hind paw in NpHR‐ and eYFP‐ expressing mice 2 weeks after SNI; Group: *F*_(1, 18)_ = 0.70, *P* = 0.42; Light on versus off, *F*_(2, 36)_ = 7.53, *P* = 0.0019 for F); Group: *F_(_*_1, 18)_ = 9.97, *P* = 0.0054 for G); *n* = 10 in each group. H–M) Example heat maps (H, J, and L) and quantification of % time spent in light‐paired chamber during pretest and test sessions (I, K, and M) in ChR2, eYFP, and NpHR mice; *t* = 2.74, *P* = 0.023 for I); *t* = 2.19, *P *= 0.056 for K); *t* = 2.47, *P* = 0.036 for M); *n *= 10 in each group. N–P) Quantification of depression‐like behaviors in three groups of mice; *F*_(2, 27)_ = 40.41, *P* < 0.0001, *n *= 10 in each group for N); *F*_(2, 27)_ = 33.35, *P* < 0.0001, *n* = 10 in each group for O); *F*_(2, 21)_ = 19.97, *P *< 0.0001, *n* = 8 in each group for P). *P < 0.05. **P < 0.01; Two‐way repeated measures ANOVAs with Tukey's post‐hoc analysis for panels D–G; One‐way ANOVAs with Tukey's post‐hoc analysis for panels N–P; Two‐tailed paired *t*‐test for panels I, K and M. Scale bars: 100 µm.

To examined whether LC‐GABA neurons modulate pain‐associated behaviors via projections to the vlPAG, we transfected ChR2, NpHR, or eYFP into LC‐GABA neurons with viral vectors and placed an optical fiber above the ipsilateral vlPAG to enable modulation of the LC^GABA^‐vlPAG pathway (**Figure** **8**A,B; Figure S12A–C, Supporting Information). We found that optogenetic activation or inhibition of the LC GABA terminals in the vlPAG with blue light (473 nm, 5 ms, 20 Hz, 5 mW) or yellow light (589 nm, 2 min, 3 mW) elicited a significant increase or decrease in PWT and PWL on both hind paws (Figure S12D–K, Supporting Information), induced CPP or CPA (Figure S12L–O, Supporting Information), and alleviated or exacerbated depression‐like behaviors (immobility time in the TST and FST, and sucrose preference in the SPT) in naïve mice (Figure S12P–R, Supporting Information).

We further investigated the behavioral outcomes in SNI mice following the modulation of the LC^GABA^‐vlPAG pathway (Figure 8A–C). Similar to the activation of LC‐GABA neurons in naïve mice, the optogenetic activation of the LC^GABA^‐vlPAG projection led to an increase in PWT and PWL (Figure 8D,E), CPP to the light‐paired chamber (Figure 8H–K), and antidepressant effects in the TST, FST and SPT (Figure 8N–P). On the contrary, inhibition of the LC^GABA^‐vlPAG pathway exacerbated hyperalgesia (manifested as reduced pain thresholds) (Figure 8F,G), caused CPA (Figure 8J–M), and prolonged immobility time in the TST and FST and compromised sucrose preference in the SPT (Figure 8N–P). These data demonstrate that the optogenetic modulation of the LC^GABA^‐vlPAG pathway effectively mimicked the behavioral changes induced by optogenetic modulation of LC‐GABA neurons in both naïve and SNI mice.

To further investigate whether LC‐GABA neurons modulate pain and comorbid depression via vlPAG‐GABA or vlPAG‐Glu neurons, we selectively inhibited these neurons with chemogenetic technique, while we stimulated the LC^GABA^‐vlPAG projection with optogenetic technique (Figure S13A,N, Supporting Information). In either naïve or SNI mice, CNO inhibition of vlPAG‐GABA neurons significantly increased PWT and PWL (Figure S13B–G, Supporting Information). Combining CNO‐mediated inhibition of vlPAG‐GABA neurons with optogenetic activation of the LC^GABA^‐vlPAG pathway did not further alter PWT or PWL compared to chemogenetic inhibition of vlPAG‐GABA neurons alone. In SNI mice, similar to optogenetic stimulation of the LC^GABA^‐vlPAG projection, chemogenetic inhibition of vlPAG‐GABA neurons alone established significant CPP and produced antidepression‐like effects in the TST, FST, and SPT (Figure S13I,K–M, Supporting Information). The combination of CNO‐mediated inhibition of vlPAG‐GABA neurons with ChR2 activation of the LC ^GABA^‐vlPAG pathway produced effects on pain and depression‐like behaviors, similar to either modulation alone (Figure S13F–M, Supporting Information). These data suggest that inhibition of vlPAG‐GABA neurons mimicked and blocked analgesic and anti‐depression‐like effects of stimulation of the LC^GABA^‐vlPAG projection.

In contrast, in naïve mice, CNO‐mediated vlPAG‐Glu inhibition did not affect PWT or PWL (Figure S13O–R, Supporting Information). Similarly, a combination of CNO‐mediated vlPAG‐Glu inhibition with ChR2 activation of the LC^GABA^‐pathway did not produce any additional changes in PWT or PWL, although ChR2 activation of the LC^GABA^‐pathway alone significantly increased PWT and PWL (Figure S13O–R, Supporting Information). These data suggest that inhibition of vlPAG‐Glu neurons blocked the analgesic effect of LC‐GABA neurons.

The results indicate that the LC^GABA^‐vlPAG projection modulates pain and depression‐like behavior probably through the LC^GABA^‐vlPAG^GABA^‐vlPAG^Glu^ pathway.

## 3 Discussion

In this study, we explored the role of LC‐GABA neurons and their circuitry in neuropathic pain and depression‐like behaviors. We found that optogenetic activation of LC‐GABA neurons elicited analgesic and anti‐depressant‐like effects in both naïve and neuropathic pain mice, while inhibition caused hyperalgesia and worsened depression‐like behaviors. Although LC‐GABA neurons innervate LC‐NA neurons^[^ ^5a^ ^]^ and attenuated their pain responses, the LC^GABA^‐LC^NA^ projection did not contribute to the pain modulation by LC‐GABA neurons, suggesting LC‐GABA and NA neurons independently regulate pain. We identified the LC^GABA^‐vlPAG projection mediating LC‐GABA neuron effects on pain and depression‐like behaviors. Notably, in SNI mice, LC‐GABA neurons were hyperactive, with enhanced LC^GABA^‐vlPAG projections. Neuromodulation experiments showed that stimulating LC‐GABA neurons or the LC^GABA^‐vlPAG projection mitigated pain hypersensitivity and depression‐like behaviors in SNI mice. Therefore, the LC^GABA^‐vlPAG pathway may underlie the pathophysiology of neuropathic pain and serve as a potential therapeutic target for comorbid pain and depression.

The LC‐NA system provides endogenous noradrenergic inhibition of the spinal cord through a descending pathway.^[^ ^7^, ^9^ ^]^ Lesion or silencing of LC‐NA neurons exacerbates hyperalgesia in both inflammatory and neuropathic pain models,^[^ ^4^, ^10^ ^]^ whereas activation of LC‐NA projection to the spinal cord attenuated sensory hypersensitivity.^[^ ^4b^ ^]^ Consistently, we observed elevated pain thresholds in both naïve and SNI mice following optogenetic activation of LC‐NA neurons, while ablation of LC‐NA neurons reduced mechanical and thermal pain thresholds. These results align with previous studies using chemogenetic modulation of LC‐NA neurons and pharmacological regulation of the LC.^[^ ^4^, ^11^ ^]^ Although LC‐GABA neurons suppressed pain responses of LC‐NA neurons, their analgesic effect may not depend on LC‐NA neurons. For instance, the ablation of LC‐NA neurons did not eliminate antinociceptive effects induced by LC‐GABA neuron activation in both naïve and SNI mice. In SNI mice, simultaneous activation of LC‐GABA and LC‐NA neurons produced stronger analgesic effects than activation of either LC‐GABA or LC‐NA neurons alone. These findings suggest that while LC‐GABA neurons innervate LC‐NA neurons, they may function independently in pain modulation.

Activation of LC‐NA and LC‐GABA neurons had similar effects on pain thresholds and depression‐like behaviors, but not on rewarding‐like behavior, in naïve mice. Specifically, the activation of LC‐NA neurons had an antidepressant effect, possibly by enhancing mood regulation pathways and promoting positive affect.^[^ ^12^ ^]^ In SNI mice, activation of LC NA and LC‐GABA neurons conferred analgesic and rewarding effects, but had opposing effects on depression‐like behaviors, with LC‐NA activation worsening and LC‐GABA activation alleviating these behaviors. Notably, LC‐NA neuron activation established CPP (probably due to its analgesic effect) but worsened depression‐like behaviors, probably involving dysregulation of mood circuits in the context of chronic pain.^[^ ^3^, ^11^ ^]^ Importantly, ablation and optogenetic inhibition of LC‐NA neurons did not eliminate analgesic, rewarding, and anti‐depressant effects of LC‐GABA neuron activation, further supporting that LC‐NA and LC‐GABA neurons regulate these behaviors through different mechanisms.

We also observed that activation of LC‐NA neurons differentially affected analgesic, rewarding, and antidepressant‐like effects induced by LC‐GABA neuron activation depending on physiological and or neuropathic pain conditions. As for the analgesic effect, we observed that in sham mice, simultaneous stimulation of LC GABA and LC‐NA neurons elevated thermal, but not mechanical, threshold more dramatically than stimulation of either LC‐GABA neurons or NA neurons; in SNI mice, simultaneous stimulation of LC‐GABA and LC‐NA neurons elevated both thermal and mechanical thresholds more dramatically than stimulation of LC‐GABA or NA neurons alone. Activation of LC‐NA neurons eliminated the rewarding effect of LC‐GABA neuron stimulation in naïve mice, but not in SNI mice, and reversed the antidepression‐like effect of LC‐GABA neuron stimulation in both naïve and SNI mice. Therefore, we propose that the downstream pathways of LC‐NA and LC‐GABA neurons may interact to regulate pain, reward, and depression‐like behaviors, and the outcomes may be influenced by neural plasticity in neuropathic pain.

Within the vlPAG, both GABA and Glu neurons play important roles in pain modulation and emotional processing.^[^ ^8a,c^ ^]^ Specifically, vlPAG‐GABA neurons contribute to nociception by inhibiting the descending pathway.^[^ ^8d^ ^]^ Conversely, activation of vlPAG‐Glu neurons exerts analgesic effects.^[^ ^13^ ^]^ We demonstrated that LC‐GABA neurons formed inhibitory synapses onto both GABA and Glu neurons in the vlPAG with a relatively weaker synaptic connection with Glu neurons. As vlPAG‐Glu neurons receive dense inhibitory inputs from local GABA neurons,^[^ ^8^, ^14^ ^]^ it is possible that for vlPAG‐Glu neurons, the inhibition from LC‐GABA neurons may be weaker than that from local GABA neurons. Following this, stimulation of LC‐GABA neurons may cause disinhibition in vlPAG‐Glu neurons via inhibiting vlPAG‐GABA interneurons. This assumption may explain our electrophysiological observation that optogenetic stimulation of the LC^GABA^‐vlPAG projection silenced vlPAG‐GABA neurons, but increased firing rate in vlPAG‐Glu neurons. This disinhibitory mechanism (LC^GABA^‐vlPAG^GABA^‐vlPAG^Glu^) may be involved in the analgesic effects of LC^GABA^‐vlPAG projection in naïve and SNI mice because it has been demonstrated that direct stimulation of vlPAG‐Glu neurons confers analgesic effect.^[^ ^13^ ^]^ Combining optogenetic stimulation of the LC^GABA^‐vlPAG projection and chemogenetic inhibition of either vlPAG GABA or Glu neurons in naïve mice, we found that the analgesic effect of the LC^GABA^‐vlPAG projection was mimicked by inhibition of vlPAG‐GABA neurons and blocked by inhibition of either vlPAG‐GABA or Glu neurons. The LC^GABA^‐vlPAG^GABA^‐vlPAG^Glu^ pathway may also contribute to the effects of the LC^GABA^‐vlPAG projection on depression‐like behaviors in SNI mice. We observed that stimulation or inhibition of the LC^GABA^‐vlPAG projection mitigated or exacerbated depression‐like behaviors in SNI mice; the antidepression‐like effect of the LC^GABA^‐vlPAG projection activation was mimicked and blocked by the inhibition of vlPAG‐GABA neurons.

Surprisingly, we observed that SNI mice exhibited hyperactivity in LC‐GABA neurons, enhanced GABA release onto vlPAG‐GABA neurons, which is followed by hypoactivity in vlPAG‐GABA neurons, but decreased GABA release onto vlPAG‐Glu neurons, accompanied by hyperactivity in vlPAG‐Glu neurons. The enhanced excitability of LC‐GABA neurons in SNI mice may explain why these neurons responded more strongly to pain and aversive stimulation in SNI mice. We argue that enhancement of the LC^GABA^‐vlPAG pathway may be a compensatory mechanism to limit the hyperalgesia and depression‐like behaviors; but the compensation may not be adequate to restore normal pain thresholds in neuropathic pain, and additional activation of this pathway is necessary to relieve pain and depression‐like behavior. Indeed, stimulation of LC‐GABA neurons and the LC^GABA^‐vlPAG projection relieved pain and depression‐like behaviors, including improvement of sucrose preference. These data suggest that these neuromodulation strategies may have the potential to alleviate anhedonia, a core symptom relevant to the treatment challenges and relapse susceptibility in major depression.^[^ ^15^ ^]^ In terms of pathophysiology of depression‐like behaviors, impairment of Glu transmission in the vlPAG is linked to chronic depression‐like behaviors induced by chronic stress,^[^ ^13^, ^16^ ^]^ which involves the control of dopaminergic neurons in the ventral tegmental area (VTA).^[^ ^17^ ^]^ Stimulation of the LC^GABA^‐vlPAG projection may disinhibit vlPAG‐Glu neurons, enhance the activity of VTA dopaminergic neurons. We observed that stimulation of LC‐GABA neurons and the LC^GABA^‐vlPAG projection established CPP in naïve mice, suggesting the involvement of this circuitry in reward processing. The projection from vlPAG to the VTA may play a significant role in mediating the rewarding effect. Our findings highlight the importance of this disinhibitory mechanism in regulating pain, depression‐like behaviors, and reward processing.

In this study, we observed that the efficacy of neuromodulation is associated with tools and circuits being recruited. For example, a stronger analgesic effect was obtained with optogenetic stimulation of LC‐GABA neurons (Figure 3), compared to chemogenetic stimulation of LC‐GABA neurons (Figures 5 and 6) and optogenetic stimulation of the LC^GABA^‐vlPAG projection (Figure 8). This may hint that optogenetic stimulation with higher temporal resolution has an advantage over chemogenetic stimulation in these behavioral paradigms; the LC^GABA^‐vlPAG pathway may partially mediate the role of LC‐GABA neurons in modulating pain and depression‐like behaviors.

In summary, we demonstrate that LC‐GABA neurons responded to noxious stimuli and modulated pain‐ and depression‐like behaviors. As downstream neurons of LC‐GABA neurons, LC‐NA neurons modulated pain and depression with various outcomes, depending on pain modality, behavioral phenotypes, and pain states. In contrast, stimulation of the LC^GABA^‐vlPAG projection mimicked the effects LC‐GABA neurons on pain and depression‐like behaviors. These findings advance our understanding of the role of LC‐GABA and LC‐NA neurons in comorbid neuropathic pain and depression and highlight the LC^GABA^‐vlPAG projection as a potential therapeutic target for treating comorbid pain and depression.

## 4 Experimental Section

Animal care and procedures (No. 202207S123) used in this study were approved by the Institutional Animal Care and Use Committee and the Office of Laboratory Animal Resources of Xuzhou Medical University under the Regulations for the Administration of Affairs Concerning Experimental Animals (1988) in China. All mice were maintained in the animal facility of Xuzhou Medical University. They were housed at 21 to 22 °C on a 12‐hour light/dark cycle with standard pellet chow and water ad libitum. Strains of mice used in this study were described previously: DBH‐Cre (Jackson Laboratory, stock No. 03 3951), Vgat‐Cre (Jackson Laboratory, stock No. 01 7535), and wild‐type C57BL6 mice. Male mice were used in behavioral tests, and both male and female mice were used roughly equal numbers for viral tracing experiments. Mice were at least 6 weeks old before surgery.

### Viral Vectors

The viral vectors purchased from Brain VTA (Wuhan, China) include AAV‐EF1α‐DIO‐hChR2(H134R)‐eYFP, AAV‐EF1α‐DIO‐NpHR‐eYFP, AAV‐EF1α‐DIO‐eYFP, AAV‐EF1α‐DIO‐GCaMP6s, AAV‐GAD67‐hM3Dq‐mCherry, AAV‐GAD67‐mCherry, AAV‐EF1α‐Flex‐taCasp3‐eYFP, and AAV‐hSyn‐DIO‐mGFP‐synaptophysin‐mRuby. The titter of the virus was between 2 × 10^12^–5 × 10^12^ viral genome per ml.

### Stereotaxic Surgery

Stereotaxic surgery for the injection of viral vectors to brain regions and for optical fiber implantation was performed as previously described.^[^ ^18^ ^]^ Mice were anesthetized with isoflurane (3% for anesthesia induction, 1.5% for maintenance of anesthesia). After confirmation of the absence of the tail pinch reflex, mice were stabilized in a stereotaxic apparatus (RWD Life Science Co., Ltd, Shenzhen, China) with a heating pad and their skin over the cranium was incised. Viral vectors were delivered unilaterally at a speed of 50 nl^−1^ min^−1^ with a Hamilton micro‐syringe (10 µl) controlled by a syringe pump (KD Scientific, Holliston, MA, USA or World Precision Instruments, Sarasota, FL, USA). Coordinates (mm relative to bregma) for injection were as follows: AP ‐5.35, ML ± 1, and DV −4 (for the LC); AP −4.6, ML ± 0.75, and DV −2.75 (for the vlPAG). For optogenetic experiments, an optical fiber (200 µm in diameter, NA 0.37) (Inper, Hangzhou, China) was placed above the LC or vlPAG. For fiber photometry recording, an optical fiber (200 µm in diameter, NA 0.37) (Inper, Hangzhou, China) was placed in the LC or vlPAG.

For postoperative pain relief, Meloxicam (4 mg per kg) (Aladdin Biochemical Technology, Shanghai, China) was added into drinking water for 3 days.

### Spared Nerve Injury (SNI)

A neuropathic pain mouse model was established with SNI of the sciatic nerve following a standard protocol.^[^ ^19^ ^]^ Under isoflurane anesthesia, the surgical area was prepared, and the sciatic nerve and its branches were exposed. By ligating and cutting the common peroneal and tibial nerves, the injury was created. Mice with their sciatic nerves exposed but intact were served as sham controls. Mice after surgery were placed on a heating pad till emergence from general anesthesia.

### von Frey Filament Test

Individual mice were acclimatized for at least 1 h in a test compartment on a wide gauge wire mesh supported by an elevated platform. The von Frey filaments with fiber force between 0.01–2 g were used to measure mechanical PWT of both hind paws. The 50% threshold was determined with the up–down method.^[^ ^19^, ^20^ ^]^

### Thermal Nociception Threshold

Individual mice were acclimatized for at least 1 h in a test compartment on a glass surface. The Hargreaves test was performed to measure the thermal PWL. The radiant heat was applied to the hind paw with a light source (Boerni, Tianjin, China) and recorded the latency to paw withdrawal. Three replicates were acquired, and values were averaged per hind paw per mouse.^[^ ^19^, ^21^ ^]^

### Conditioned Place Preference (CPP) Test

The CPP test was conducted in a specially designed two‐chamber box. The chambers differed in wall patterns and floor textures. The mice first underwent a 2‐day pre‐conditioning session (pretest) in which they had free access to two chambers of a CPP box for 20 min. This was followed by a 3‐day conditioning session in which blue light (20 Hz, 5 ms, 4 mW, 120 s on 60 s off, for 30 min) or yellow light (120 s constant 3 mW light with 60 s intervals for 30 min) stimulation was applied when the mice were placed in one of the two chambers for 30 min in the afternoon, whereas no stimulation was applied when the mice were placed in the opposing chamber for 30 min in the morning. Twenty‐four hours after the last conditioning session, the mice were subjected to a test session in which they were free to enter either chamber, and their time spent in each chamber was recorded. The time spent in the preferred side was calculated, excluding mice spending over 75% of their time in one chamber during the pretest session.

### Tail Suspension Test (TST) and Forced Swim Test (FST)

In the TST, each mouse was suspended by taping its tail onto a horizontal bar 50 cm above the floor and allowed to hang undisturbed for 6 min. In the FST, each mouse was placed in a 1‐liter cylinder glass beaker filled with 26 °C water for 6 min. The movement of the mice was video‐recorded and immobility time in the last 5 min in the TST and FST was used to evaluate depression‐like behavior.

### Sucrose Preference Test (SPT)

All mice were single‐housed for 1 week and then underwent adaptive training from day 1 to day 4. Mice had access to two bottles of water available on days 1 and 2, two bottles of 1% sucrose on day 3, and one bottle of water and one bottle of 1% sucrose on day 4. Following 12 h deprivation of food and water, each mouse was provided with two 200 ml bottles filled with either water or 1% sucrose solution. 1 h later, the bottles were switched and the consumption of water and sucrose was recorded after another hour. Sucrose preference was calculated as: sucrose preference percentage (%) = sucrose solution consumption (ml)/(sucrose solution consumption (ml) + water consumption (ml)) × 100%.

### Intrathecal Injection

For intrathecal drug injection, drugs (15 µl) were administrated using an insulin syringe (30 gauge). The needle of a syringe was angled slightly toward to the head and inserted in the midline at the L4‐L5 intervertebral space. Correct placement of the syringe was confirmed by a tail flick response or a retraction of the leg. Pain sensitivity was measured 15 min after injection.

### Brain Slice Electrophysiology

Brain slice electrophysiological recording was conducted following established protocols.^[^ ^18^, ^19^, ^22^ ^]^ Coronal slices (250 to 300 µm thick) containing the LC or PAG were prepared using a vibratome (Leica VT‐1200S, Nussloch, Germany) in an ice‐cold cutting solution saturated with 95% O_2_/5% CO_2_ (carbogen), containing (in mM) 85 NaCl, 75 sucrose, 2.5 KCl, 1.25 NaH_2_PO_4_, 4.0 MgCl_2_, 0.5 CaCl_2_, 24 NaHCO_3_, and 25 glucose. The brain slices were transferred into carbogenated cutting solution at 32 °C for 60 min recovery. The slices were then transferred to normal carbogenated ACSF, containing (mm) 125 NaCl, 2.5 KCl, 1.2 NaH_2_PO_4_, 1.2 MgCl_2_, 2.4 CaCl_2_, 26 NaHCO_3_, and 11 glucose, at 26 °C for at least 30 min before experiments.

Neurons in brain slices were visualized under an upright microscope (FN‐1, Nikon, Tokyo, Japan), equipped with a CCD‐camera (Flash 4.0 LTE, Hamamatsu, Hamamatsu city, Japan). Whole‐cell patch‐clamp recording was made using a patch‐clamp setup composed of a dual‐channel MultiClamp 700B amplifier, a Digidata 1550B analog‐to‐digital converter, and pClamp 10.7 software (Molecular Devices, San Jose, CA, USA). The patch electrodes had a resistance of 4–6 MΩ when filled with a low‐chloride intrapipette solution containing (in mm) 135 K gluconate, 0.2 EGTA, 0.5 CaCl_2_, 10 HEPES, 2 Mg‐ATP, and 0.1 GTP, pH: 7.2; osmolarity: 290–300 mOsm. Neurons with a holding current larger than −50 pA and a resting membrane potential more depolarized than −40 mV were excluded from the analysis. All recordings were performed at 32 ± 1 °C.

Optogenetic activation and inhibition were achieved using blue light (460 nm, 2 mW) and yellow light (560 nm, 2 mW), respectively. The light was delivered through an optical fiber (200 µm, NA 0.37) connected to a PlexBright LED light source (Plexon Inc., Hong Kong, China). Blue light‐evoked inhibitory postsynaptic currents (eIPSCs) were recorded at −40 mV in downstream neurons of LC‐GABA neurons transfected with ChR2‐eYFP. Bicuculline (BIC, 10 µm) was bath‐applied to confirm that the currents were mediated by GABA_A_ receptors, and 4‐aminopyridine (4‐AP, 100 µm) along with tetrodotoxin (TTX, 1 µm) was applied to confirm monosynaptic connections. Neuronal firing responses were elicited by current injections (0.5 s, 20–200 pA steps with a 20 pA increment and a 15 s inter‐sweep interval) in the current‐clamp mode.

### Immunohistochemistry and Imaging

To harvest brain tissue, mice were sacrificed with CO_2_ and then transcardially perfused with 15 ml of phosphate‐buffered saline (PBS) and 15 ml of 4% paraformaldehyde (PFA) in PBS. The brain was then extracted and stored in 4% PFA for at least 8 h, and then, the brain was switched to 30% sucrose solution and stored until sank. Brains were frozen with dry ice and sectioned with Leica CM‐1950 cryostat (Nussloch, Germany) into 30 µm slices. The slices were mounted onto microscope slides and cover‐slipped with mounting medium (Meilunbio, Dalian, China). A Zeiss LSM880 confocal microscope (Oberkochen, Germany) was used to image the slices. For immunohistochemistry, brain slices were washed and placed in a blocking solution for 1 h (10% donkey serum, 0.1% Triton X‐100 dissolved in PBS), and then incubated overnight at 4 °C with the appropriate primary antibody. Then, after 3 times washing, they were incubated 2 h at room temperature with the appropriate secondary antibody.

Primary antibodies include rabbit anti‐TH antibody, IgG 1:500 (Santa Cruz), and rabbit anti‐GABA IgG, 1:500 (Sigma). Secondary antibodies include Alexa 488‐ or Alexa 555‐ or Alexa 647‐conjugated donkey anti‐rabbit IgG, 1:500 (Jackson ImmunoResearch).

### Pharmacology

For assessments of the involvement of the descending pathway in pain modulation by LC‐GABA neurons, chemogenetic stimulation of LC‐GABA neurons, intrathecal drug injection, and mechanical and thermal threshold measurement were conducted 4–5 weeks after virus injection. Yohimbine hydrochloride, ondansetron, and metergoline were purchased from MedChemExpress (Monmouth Junction, NJ, USA) and were prepared as a 1 mg ml^−1^ working solution in sterilized normal saline. On the testing day, each mouse received an intrathecal injection of either drug or saline (15 µl) 30 min after CNO administration and 30 min before behavioral tests. Intrathecal injections were performed in fully conscious mice over a period of 10 s. The dose was comparable to other microinjection techniques used in mouse models.^[^ ^6^, ^23^ ^]^

### Statistical Analysis

GraphPad Prism 8.0 was used for statistical analyses. Clampfit 10.7 (Molecular Devices) was used for the analysis of electrophysiological and fiber photometry data. Figures were prepared with Adobe Illustrator 2020. All summarized data were expressed as mean ± S.E.M. Two‐tailed paired or unpaired *t*‐tests were used for comparison of a parameter between two groups if data were normally distributed. One‐way, one‐way repeated measures, two‐way, or two‐way repeated measures ANOVAs followed by Tukey's post‐hoc analysis were used for multiple comparisons. If the equal‐variance assumptions were not valid, statistical significance was evaluated with the Mann–Whitney test or ANOVA rank tests. The mean and S.E.M, *n* (the number of animals), statistical test, and *t*, *F*, and *P* values were reported in figure legends. A value of *P* < 0.05 was considered statistically significant. The minimal number of mice used in each experiment was calculated in a priori power analysis (StatMate 2.0) and the power of each experiment was set to 0.8. The sample sizes in all experiments were larger than the minimal numbers.

## Conflict of Interest

The authors declare no conflict of interest.

## Author Contributions

Y.G., X.Z., and X‐J.L. contributed equally to this work. C.Z., C.X., and D.L.T. designed and supervised this research. C.Z. and C.X. collected, analyzed, and illustrated electrophysiological data. Y.G., X.Z., X.J.L., Y.L.S., and C.Y. performed mouse survival surgeries, morphological experiments, and behavioral tests, and managed mouse colony. C.Z., C.X., D.L.T., and Y.G. wrote the manuscript. All authors read and approved the manuscript.

## Supporting information



Supporting Information

## Data Availability

The data that support the findings of this study are available from the corresponding author upon reasonable request.
