# Supplementary material for: The Locus Coeruleus‐Periaqueductal Gray GABAergic Projection Regulates Comorbid Pain and Depression
Source: Adv Sci (Weinh). 2025 Apr 7;12(26):2503739. doi: 10.1002/advs.202503739 (PMC12245010; doi:10.1002/advs.202503739)
Supplement: Supplementary file 1 — Supporting Information [file ADVS-12-2503739-s001.docx]

**Supporting Information**

**The Locus Coeruleus-Periaqueductal Gray GABAergic Projection Regulates Comorbid Pain and Depression**

Yuan Gao#, Xue Zhang#, Xiao-Juan Liu#, Yi-Ling Sun, Cui Yin, Dong-Liang Tang*, Cheng Xiao^*^, Chunyi Zhou^*^

This file includes: Figure S1-S13

| 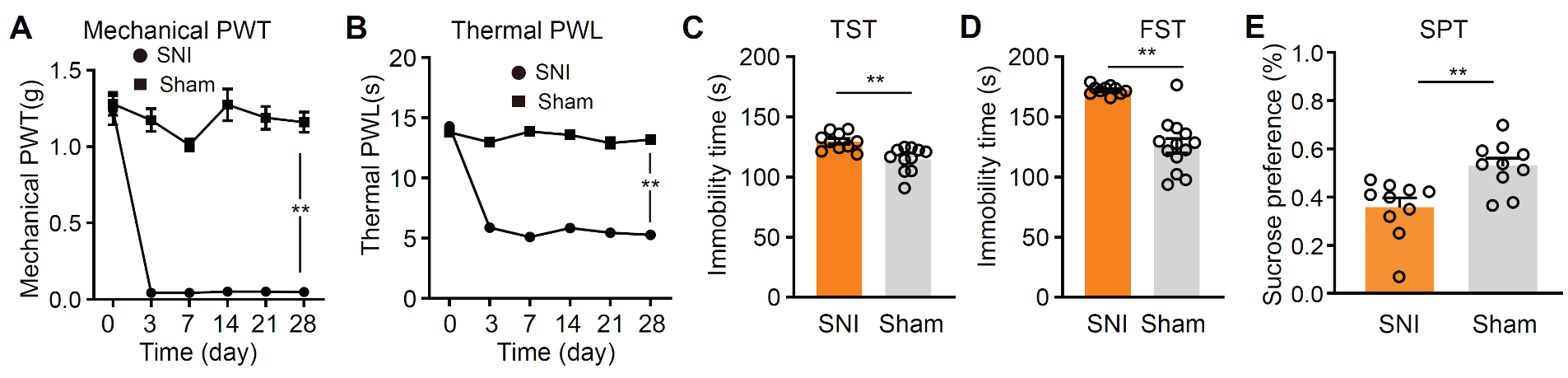 |
| --- |

**Figure S1. SNI mice develop nociceptive hypersensitivity and depression-like behavior.**

**(A, B)** Mechanical PWT and thermal PWL on the hind paw of the side that spared nerve injury (SNI) or sham surgery was performed. **(A)** Sham vs SNI: F_(1,14)_ = 772.0, P < 0.0001; **(B)** Sham vs SNI: F_(1,14)_ = 1922, P < 0.0001; n = 8 mice in each group.

**(C-E)** Quantification of depression-like behaviors in week 4-5 after SNI and sham surgery. **(C)** Immobility time of the TST, *t* = 2.54, P = 0.026, n = 6 mice in sham, n = 8 mice in SNI. **(D)** Immobility time of the FST, *t* = 3.38, P = 0.0055, n = 6 mice in sham, n = 8 mice in SNI. **(E)** Sucrose preference in the SPT, *t* = 3.45, P = 0.0029, n = 10 mice in each group. ** P < 0.01. Two-way repeated measures ANOVA in (**A**, **B**). Two-tailed paired *t*-test in (**C-E**).


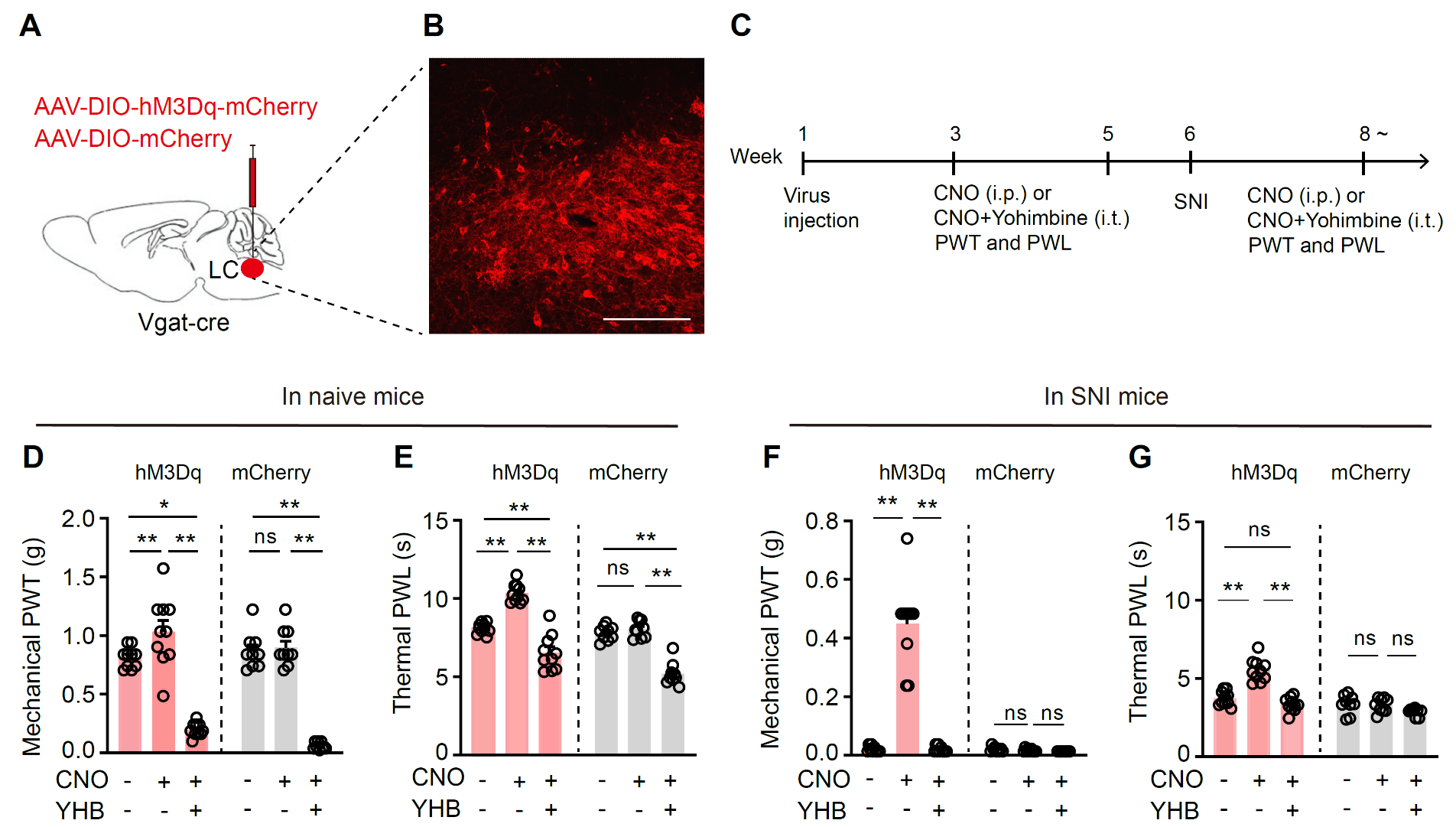


**Figure S2. Yohimbine blocks antinociceptive effects induced by chemogenetic activation of LC-GABA neurons.**

**(A)** Schematic diagram for virus injection.

**(B)** An example image showing the expression of hM3Dq-mCherry in the LC.

**(C)** Schematic diagram of experimental design.

**(D-G)** Mechanical PWT and thermal PWL in naïve and SNI mice. **(D)** Drug effect: F_(2, 51)_ = 149.0, P < 0.0001; **(E)** Drug effect: F_(2, 51)_ = 149.0, P < 0.0001; n = 10 in hM3Dq, n = 9 in mCherry. **(F)** Drug effect: F_(2, 51)_ = 78.84, P < 0.0001; **(G)** Drug effect: F_(2, 51)_ = 32.47, P < 0.0001; n = 10 in hM3Dq, n = 9 in mCherry. Two-way repeated measures ANOVA in (**D-G**). CNO: Clozapine-N-oxide. PWL: paw withdrawal latency. PWT: paw withdrawal threshold. SNI: spared nerve injury. YHB: yohimbine.


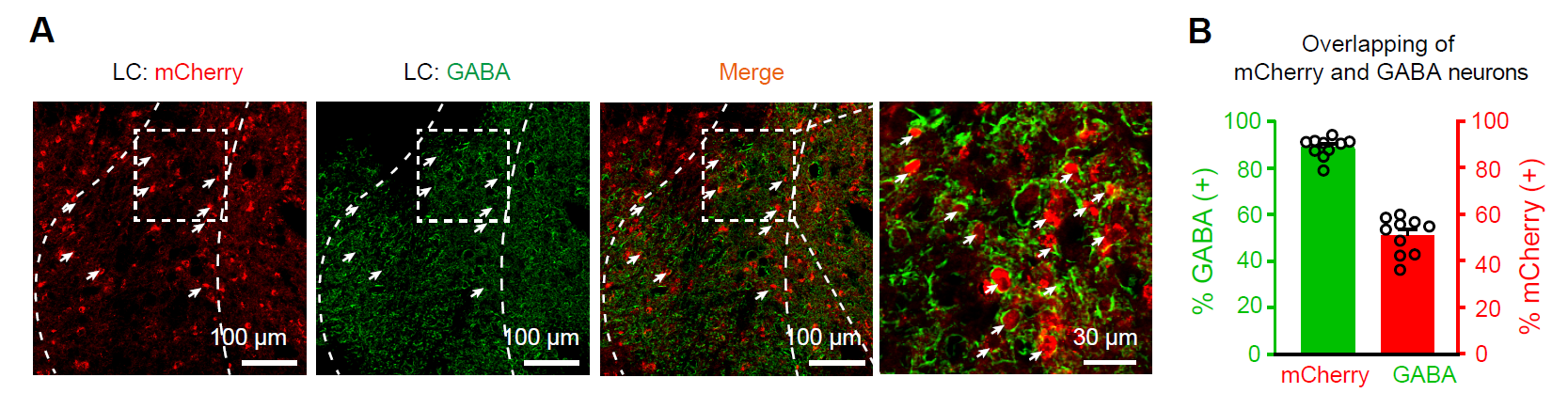
**Figure S3. Selective labeling of GABAergic neurons with viral vector in the LC.**

AAV-GAD67-hM3Dq-mCherry was injected into the LC of wild type mice. Immunofluorescent assay was conducted in 3 weeks. **(A)** Representative images showing LC neurons being labeled by mCherry (left panel), GABA antibody (middle), and merged images. **(B)** Percentages of neurons co-labeled with mCherry and GABA-antibody in mCherry- and GABA-labeled neurons. n = 10 sections from 3 mice.


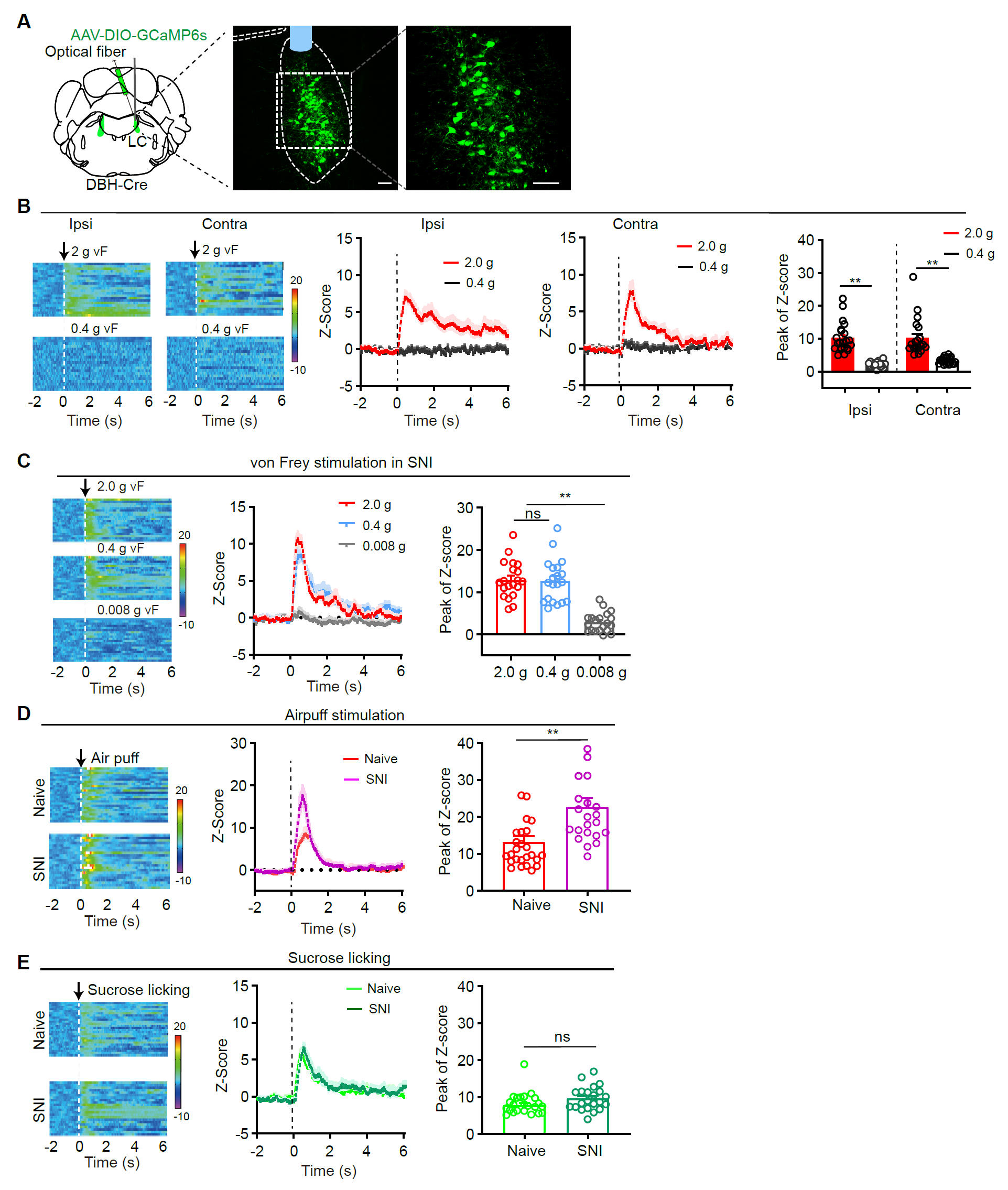
**Figure S4. LC-NA neurons respond to pain-like stimuli and emotional changes.** Fiber photometry recording of GCaMP6s signals in LC-NA neurons in response to various stimuli, including von Frey filament and thermal stimulation on the hind paws, air puff on one side of the face, and sucrose licking behavior in naïve and SNI mice. **(A)** DBH-Cre mice were injected with AAV-EF1α-DIO-GCaMP6s in the LC. Images of a coronal section shows GCaMP6s expressing-NA neurons in the LC. (**B**) Heat maps (Left panels), averaged normalized traces (Middle panels)**,** and summary (Right panels) show changes in GCaMP6s signals in LC-NA neurons in response to suprathreshold (2 g) or subthreshold (0.4 g) von Frey filament stimulation or thermal stimulation on hind paws in naïve mice. n = 5 mice in each group (Right, F_(3, 79)_ = 28.45, P < 0.0001).

**(C)** Heat maps (Left panels), averaged normalized traces (Middle panels)**,** and summary (Right panels) show changes in GCaMP6s signals in LC-NA neurons in response to suprathreshold (2 g and 0.4 g) or subthreshold (0.008 g) von Frey filament stimulation on SNI injured hind paws in SNI mice. n = 5 mice in each group (Right, F_(2, 57)_ = 39.54, P < 0.0001).

**(D)** Heat maps (Left panels), averaged normalized traces (Middle panels) show changes in GCaMP6s signals in LC-NA neurons in response to air puff stimulation on one side of the face in naïve and SNI mice (Right, t = 3.24, P = 0.0022).

**(E)** Heat maps (Left panels), averaged normalized traces (Middle panels) showing changes in GCaMP6s signals in LC-NA neurons in response to sucrose licking behavior in naïve and SNI mice (Right, t = 1.66, P = 0.11).

** P < 0.01, One-way ANOVA with Tukey’s post-hoc analysis for (**B** and **C**); ** P < 0.01. Two-tailed unpaired *t*-test for (**D** and **E**) **;** n = 5 mice in each group. Dashed lines indicate stimulus onset. Scale bars: 100 μm. Both the Z-score and heat map color bars are in units of ΔF/F.


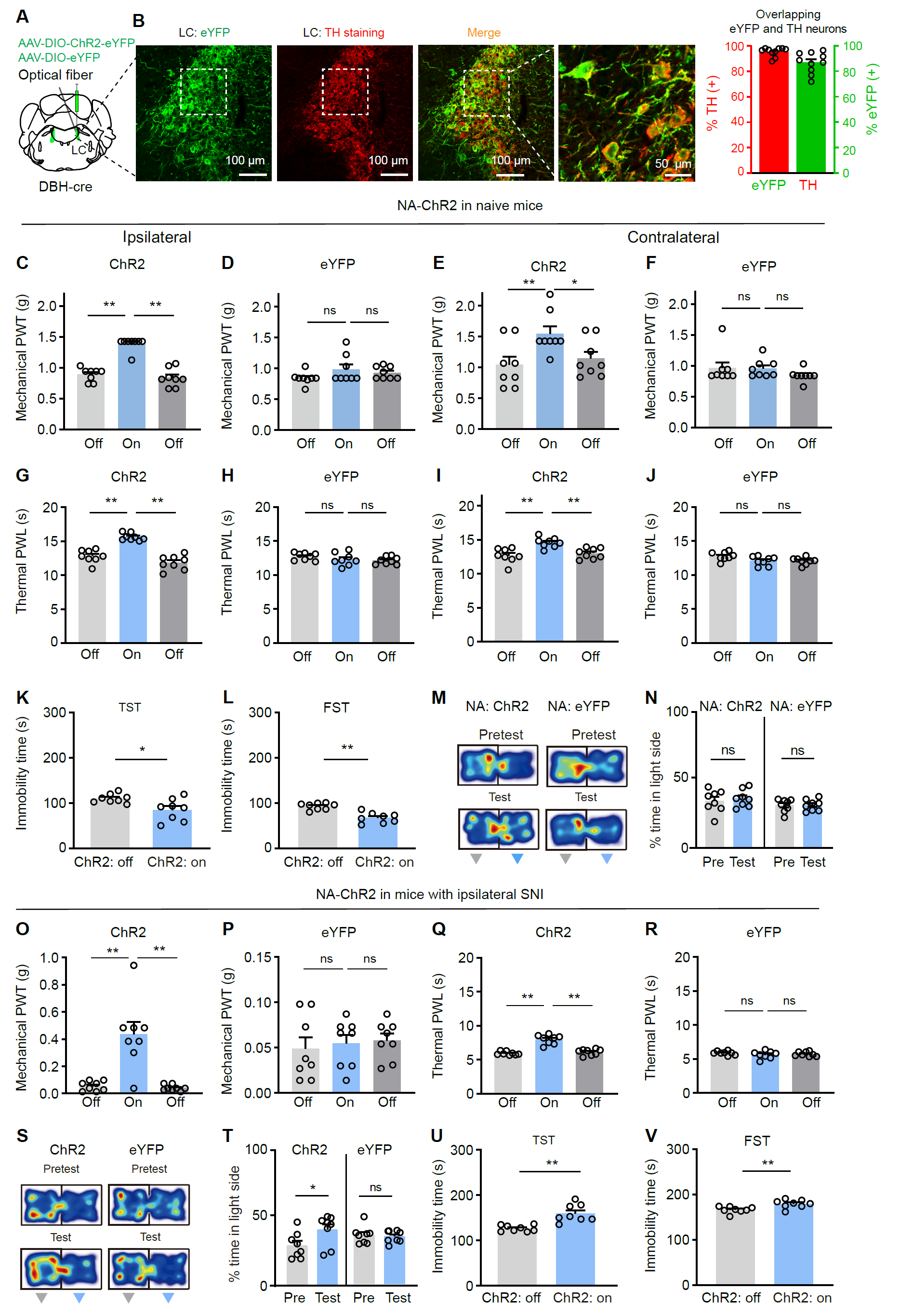
**Figure** S**5.** **Optogenetic activation of LC-NA neurons modulates pain thresholds and depression-like behaviors in naïve and SNI mice.**

**(A)** Schematic diagram for virus injection and optical fiber placement for selective activation of LC-NA neurons.

**(B)** Example images and summary showing that the viral vector specifically labeled LC NA neurons. Data were summarized from 10 sections from 3 mice.

**(C-F)** The effect of blue light illumination on mechanical PWT on hind paws of ChR2- and eYFP-mice. **(C)** Ipsilateral PWT in ChR2 mice, F_(2, 14)_ = 40.53, P < 0.0001; **(D)** Ipsilateral PWT in eYFP mice, F_(2, 14)_ = 2.32, P = 0.14; **(E)** Contralateral PWT in ChR2 mice, F_(2, 14)_ = 16.87, P = 0.008; (**F)** Contralateral PWT in eYFP mice, F_(2, 14)_ = 1.65, P = 0.23; n = 8 mice in each group.

**(G-J)** The effect of blue light illumination on thermal PWL on hind paws of ChR2- and eYFP-mice. **(G)** Ipsilateral PWL in ChR2 mice, F_(2, 14)_ = 55.42, P < 0.0001; **(H)** Ipsilateral PWL in eYFP mice, F_(2, 14)_ = 2.70, P = 0.10; **(I)** Contralateral PWL in ChR2 mice, F_(2, 14)_ = 19.7, P < 0.0001; **(J)** Contralateral PWL in eYFP mice, F_(2, 14)_ = 3.52, P = 0.058; n = 8 mice in each group.

**(K** and **L)** Quantification of depression-like behaviors before and during blue light illumination of the LC in ChR2 mice. **(K)** Immobility time in the TST, t = 2.67, P = 0.018, n = 8 in each group; **(L)** Immobility in the FST, t = 5.22, P = 0.0001; n = 8 in each group.

**(M** and **N)** Example heat maps **(M)** and quantification of % time spent in the light-paired chamber during precondition and test sessions **(N)** in ChR2 and eYFP mice. Group factor: F_(1,14)_ = 0.38, P = 0.55; n = 8 mice in each group.

**(O-R)** The effect of blue light illumination in the LC on mechanical PWT and thermal PWL in ChR2- and eYFP-mice 2 weeks post-SNI on the ipsilateral hind paw. **(O)** PWT in ChR2 mice, F_(2, 14)_ = 21.08, P < 0.0001; **(P)** PWT in eYFP mice, F_(2, 14)_ = 0.84, P = 0.45; **(Q)** PWL in ChR2 mice, F_(2, 14)_ = 44.36, P < 0.0001; **(R)** PWL in eYFP mice, F_(2, 14)_ = 1.14, P = 0.35; n = 8 mice in each group.

**(S and T)** Example heat maps **(S)** and quantification of % time spent in the light-paired chamber during precondition and test sessions (**T**) in ChR2 and eYFP mice. Group factor: F_(1,14)_ = 11.33, P = 0.0046; n = 8 in each group.

**(U and V)** Quantification of depression-like behaviors before and during blue light illumination of the LC in ChR2 mice. **(U)** Immobility time in the TST, t = 4.52, P = 0.0005, n = 8 in each group. **(V)** Immobility time in the FST, t = 3.13, P = 0.0073; n = 8 in each group.

*P < 0.05. **P < 0.01; One-way ANOVA with Tukey’s post-hoc analysis for **(C-J, O-R)**; Two-tailed paired *t*-test for **(K, L, U and V);** Two-way repeated measures ANOVA with Tukey’s post-hoc analysis for **(N and T)**. Scale bars: 100 μm.


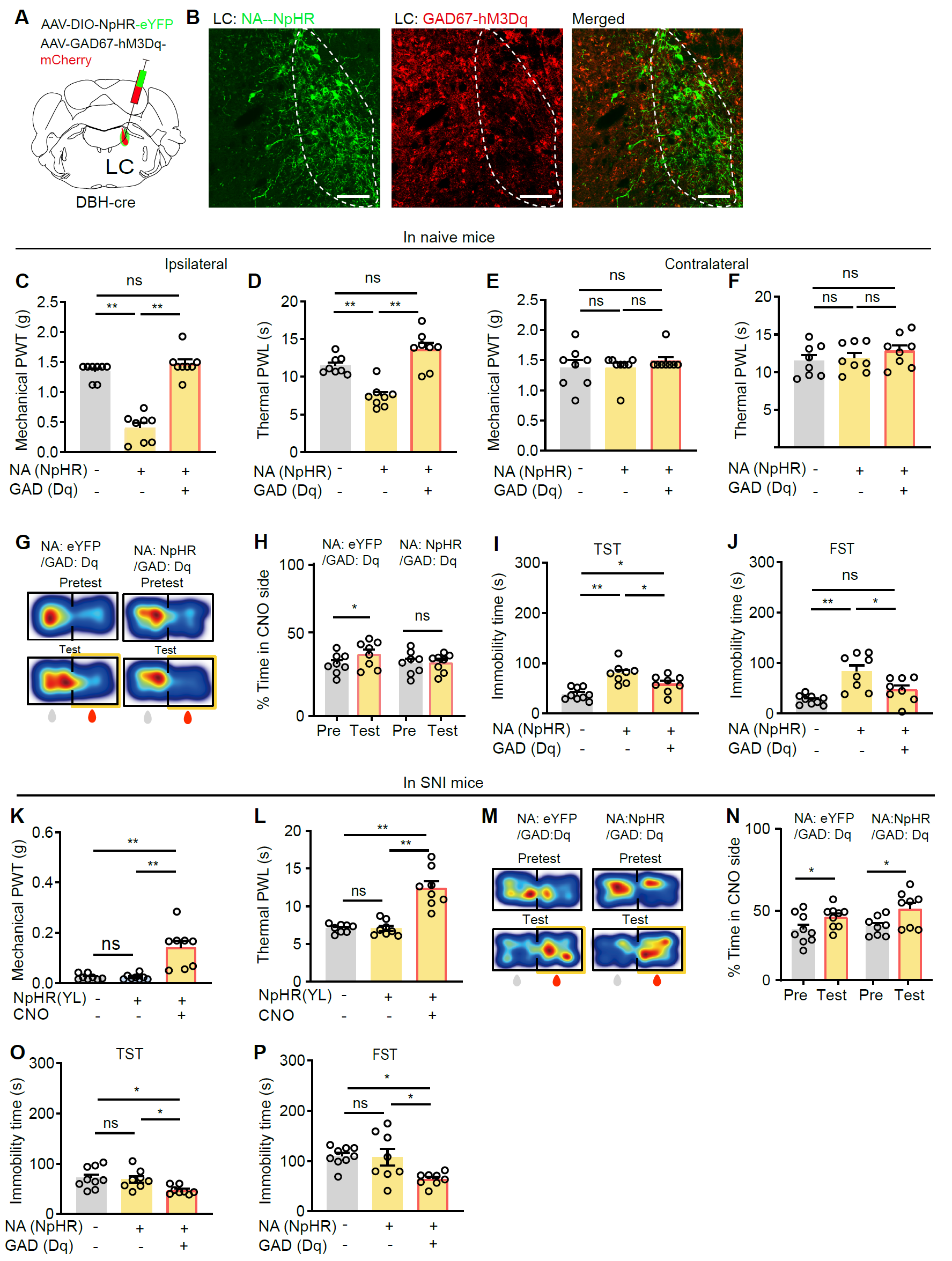
**Figure S6. Manipulation of LC-NA and LC-GABA neurons in pain thresholds and depression-like behaviors. (A)** Schematic diagram of viral injections targeting NA neurons and GABA-neurons in the LC region in DBH-Cre mice. **(B)** Example images show expression of NpHR and hM3Dq-mCherry in the LC regions.

**(C-F)** Mechanical PWT and thermal PWL in naïve mice following NpHR-mediated inhibition of LC-NA neurons alone or in combination with CNO activation of LC-GABA neurons. Both ipsilateral and contralateral responses to mechanical and thermal stimuli are shown (**C**, F_(2, 21)_ = 65.36, P < 0.0001; **D**, F_(2, 21)_ = 24.8, P < 0.0001; **E**, F_(2, 21)_ = 0.47, P = 0.63; **F**, F_(2, 21)_ = 0.90, P = 0.42; n = 8 mice in each group).

**(G-H)** Example heat maps **(G)** and quantification of % time spent in the yellow light plus CNO-paired chamber during precondition and test sessions (**H**) in NA-eYFP: GABA-hM3Dq and NA-NpHR: GABA-hM3Dq mice in a CPP test (Group: F_(1, 14)_ = 5.24, P = 0.038, n = 8 mice in each group).

**(I-J)** Immobility time in the TST and FST in naïve mice following NpHR-mediated inhibition of LC-NA neurons or in combination of CNO-activation of LC-GABA neurons (**I**, F_(2, 22)_ = 13.35, P = 0.002; **J**, F_(2, 22)_ = 10.99, P = 0.005, n = 8 mice in each group).

**(K-L)** Mechanical PWT and thermal PWL in mice subjected to ipsilateral SNI following the same manipulation as panels **(C)** (**K**, F_(2, 21)_ = 16.62, P < 0.001; **L**, F_(2, 21)_ = 130.03, P < 0.001; n = 8 mice in each group).

**(M-N)** Example heat maps **(M)** and quantification of % time spent in the yellow light plus CNO-paired chamber in the CPP test (**N**) in SNI mice following the same manipulation as panel **(G-H)** (**N**, Group factor: F_(1, 15)_ = 17.35, P = 0.008, n = 8 mice in each group).

**(O-P)** Immobility time in the TST and FST in SNI mice following the same manipulations as panels **(I-J)** (**O**, F_(2, 22)_ = 4.99, P = 0.016; **P**, F_(2, 22)_ = 5.94, P = 0.0087; n = 8 mice in each group).

*P < 0.05. **P < 0.01; One-way ANOVA with Tukey’s post-hoc analysis for **(C-F, I-L, O and P)**; Two-way repeated measures ANOVA with Tukey’s post-hoc analysis for **(H and N)**. Scale bars: 100 μm. The yellow line frame and red water drop shape in panels **G** and **M** indicate that the chamber was paired with yellow light stimulation and CNO administration.


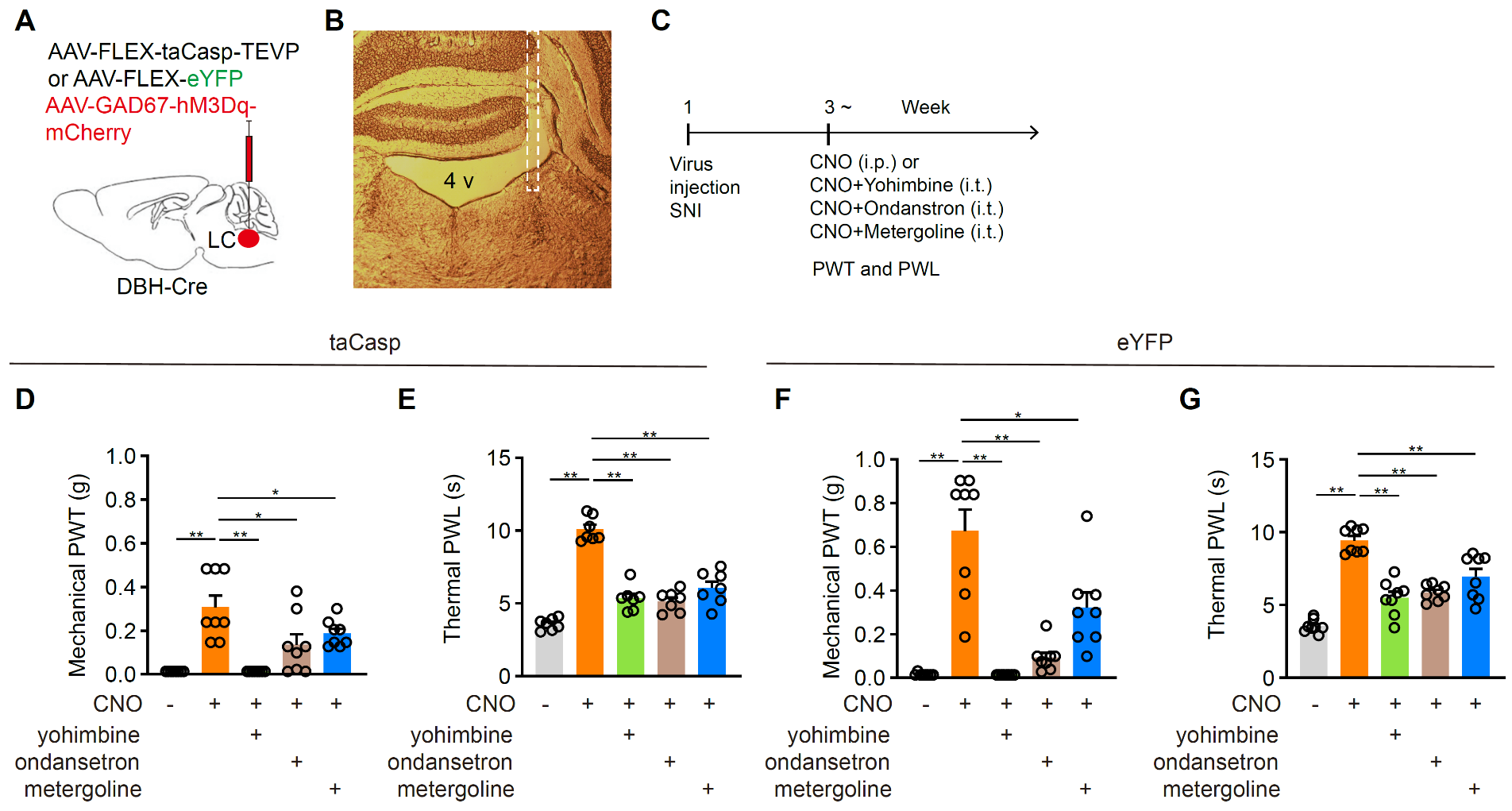
**Figure S7. Antinociceptive effects of LC-GABA neurons are mediated by spinal serotoninergic and noradrenergic receptors in mice with and without ablation of LC-NA neurons.**

**(A)** Schematic diagram for selective ablation of LC-NA neurons and chemogenetic activation of LC-GABA neurons with virus vectors.

**(B)** Example bright field image of needle track for virus injection into the LC.

**(C)** Schematic diagram of experimental design.

**(D-G)** Mechanical PWT and thermal PWL in SNI mice with intraperitoneal (*i.p.*) injection of CNO or combined with intrathecal injection (*i.t.*) of yohimbine, ondansetron, or metergoline. **(D)** PWT in mice with ablation of LC-NA neurons (taCasp), F_(4, 35)_ = 13.61, P < 0.0001, n = 8 mice in each group. **(E)** PWL in taCasp mice, F_(4, 30)_ = 59.85, P < 0.0001, n = 7 mice in each group. **(F)** PWT in mice without ablation of LC-NA neurons (eYFP), F_(4, 35)_ = 26.13, P < 0.0001, n = 8 mice in each group. **(G)** PWL in eYFP mice, F_(4, 35)_ = 37.6, P < 0.0001, n = 8 mice in each group.

*P < 0.05. **P < 0.01; One-way ANOVA with Tukey’s post-hoc analysis for **(D-G).**


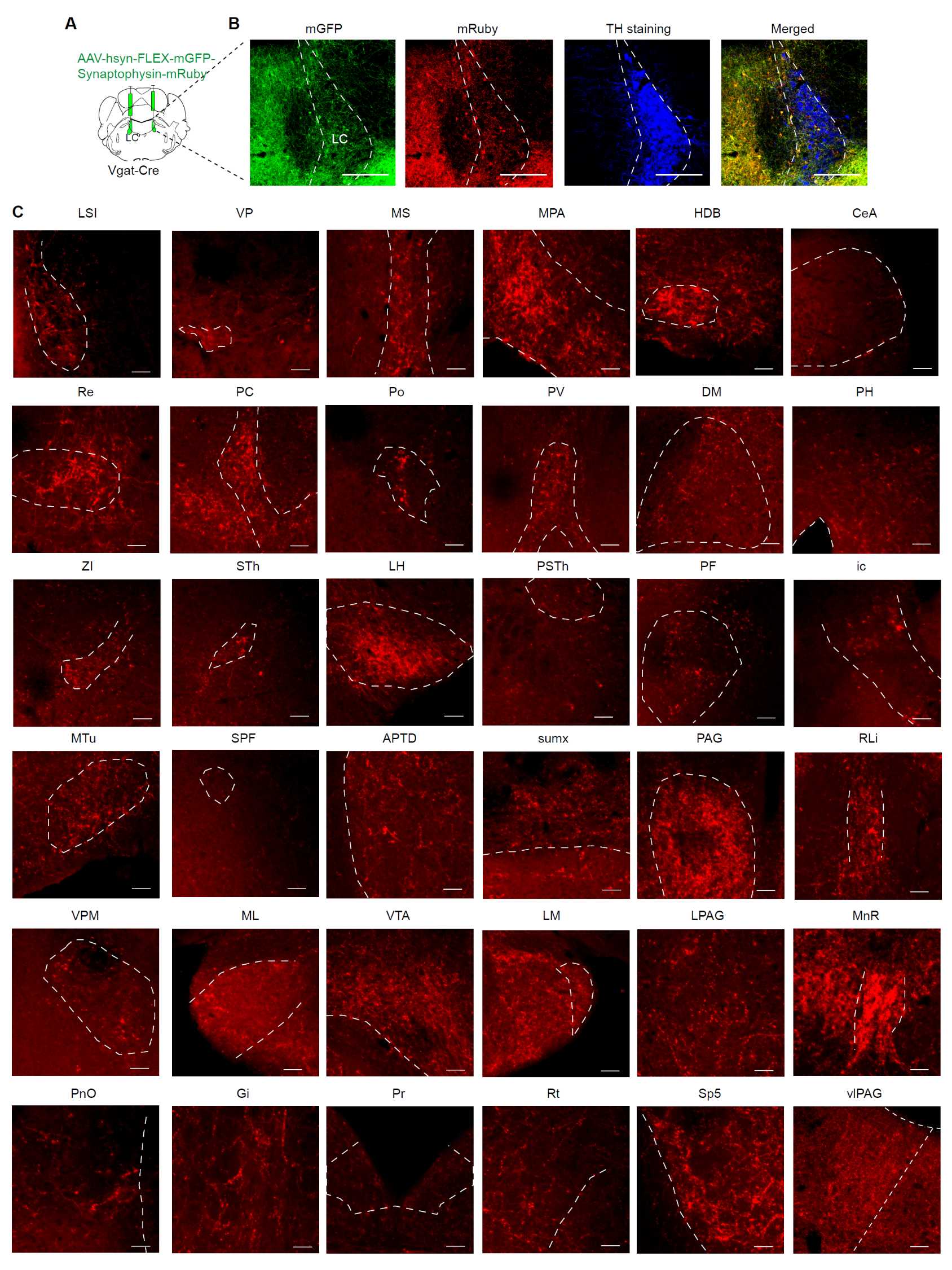
**Figure S8. Mapping of projections of LC-GABA neurons**

**(A, B)** Schematic diagram for virus injection to label axonal projections of LC-GABA neurons (**A**) and example images showing eGFP and mRuby expression in the LC area (**B**). TH-antibody-staining was used to mark location of the LC.

**(C)** Example images showing mRuby-labeled terminals in red. Scale bars: 100 µm.

APTD, anterior part, dorsal area, of the anterior pretectal nucleus; CeA, Central amygdala nucleus; DM, dorsomedial thalamic nucleus; Gi, gigantocellular reticular nucleus; HDB, horizontal limb of the diagonal band; ic, internal capsule; LH, lateral hypothalamus; LM, lateral mammillary nucleus; LPAG, lateral periaqueductal gray; LSI, lateral septal nucleus, intermediate part; ML, medial lemniscus, lateral Part; MnR, median raphe nuclei; MPA, medial prefrontal Area; MS, medial septal nucleus; MTu, medial tuberal nucleus; PAG, periaqueductal gray; PC, paracentral thalamic nucleus; PF, parafascicular nucleus; PH, posterior hypothalamic area; PnO, oral Part of the pontine reticular nucleus; Po, posterior group of the thalamic nuclei; Pr, pregeniculate nucleus; PSTh, parasubthalamic nucleus; PV, paraventricular thalamic nucleus; Re, reuniens nucleus; RLi, nucleus reticularis lateralis; Rt, reticular nucleus; Sp5, spinal trigeminal tract; SPF, subparafascicular nucleus; STh, subthalamic nucleus; SUMx, supramammillary nucleus, pars supracapsularis; VLPAG, ventrolateral periaqueductal gray; VP, ventral pallidum, ventral part; VPM, ventroposterior medial nucleus of the thalamus; VTA, ventral tegmental area; ZI, zona incerta.


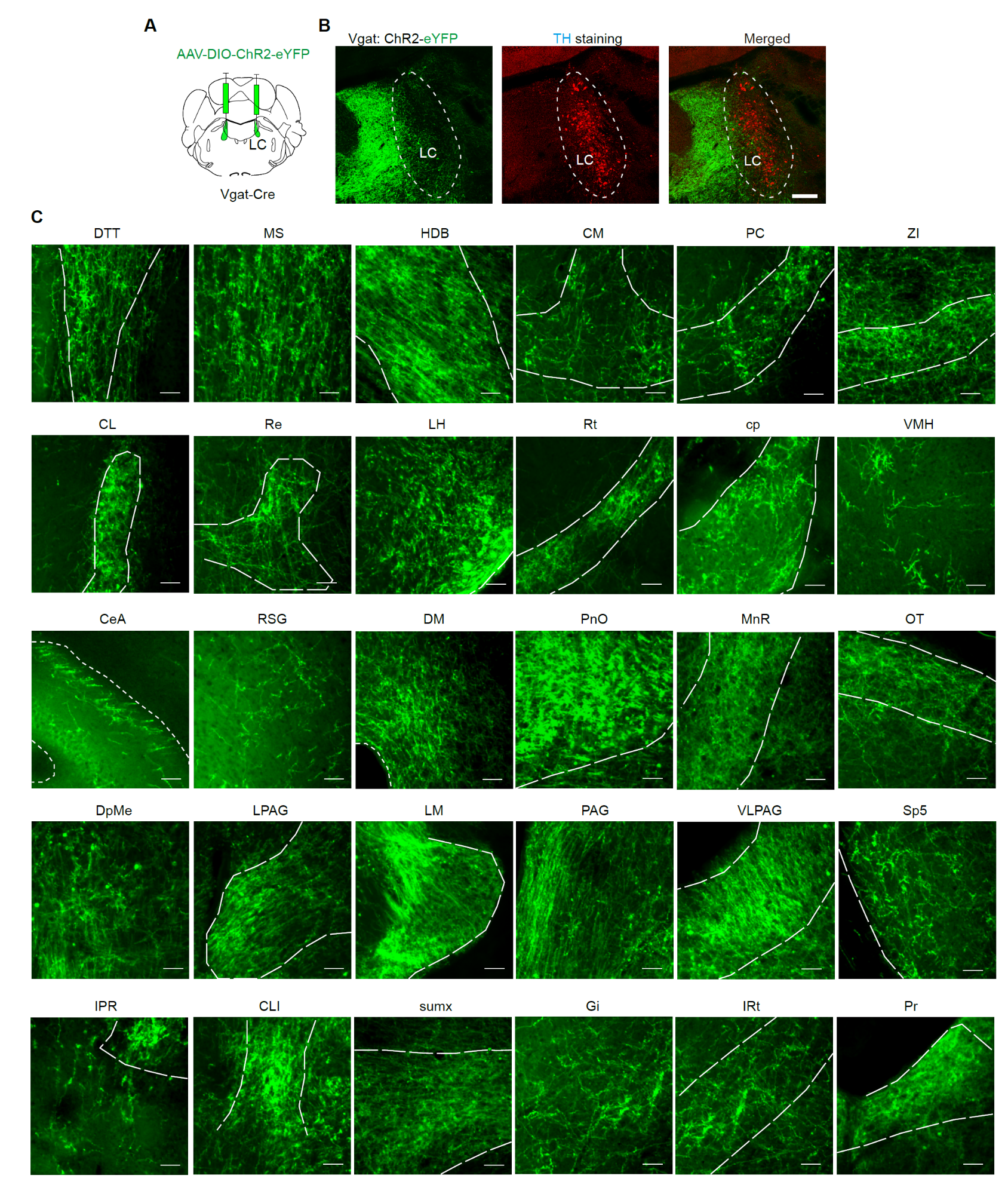
**Figure S9. Anterograde tracing of downstream projections of LC-GABA neurons.**

**(A)** Expression of ChR2-eYFP in LC-GABA neurons.

**(B)** Example images of eYFP expression in the LC and NA neurons labeled with TH-antibody-staining.

**(C)** Example images of eYFP-labeled axonal fibers from LC-GABA neurons in multiple brain regions.

Scale bars: 100 µm.

CeA, central amygdala nucleus; CL, central lateral thalamic nucleus; CLI, caudal linear nucleus; CM, central medial nucleus of the thalamus; cp, cerebral peduncle; DM, dorsomedial thalamic nucleus; DpMe, deep mesencephalic reticular nucleus; DTT, dorsal tegmental nucleus; Gi, gigantocellular reticular nucleus; HDB, horizontal limb of the diagonal band; IPR, interpeduncular nucleus, rostral subnucleus; IRt, intermediate reticular nucleus; LH, lateral hypothalamus; LM, lateral mammillary nucleus; LPAG, lateral periaqueductal gray; MnR, medial raphe nuclei; MS, medial septum; OT, optic tract nucleus; PAG, periaqueductal gray; PC, paracentral thalamic nucleus; PnO, oral part of the pontine reticular nucleus; Pr, pregeniculate nucleus; Re, reuniens nucleus; RSG, retrosplenial granular cortex; Rt, reticular nucleus; Sp5, spinal trigeminal tract; SUMx, supramammillary nucleus, pars supracapsularis; VMH, ventromedial hypothalamic nucleus; ZI, zona incerta.


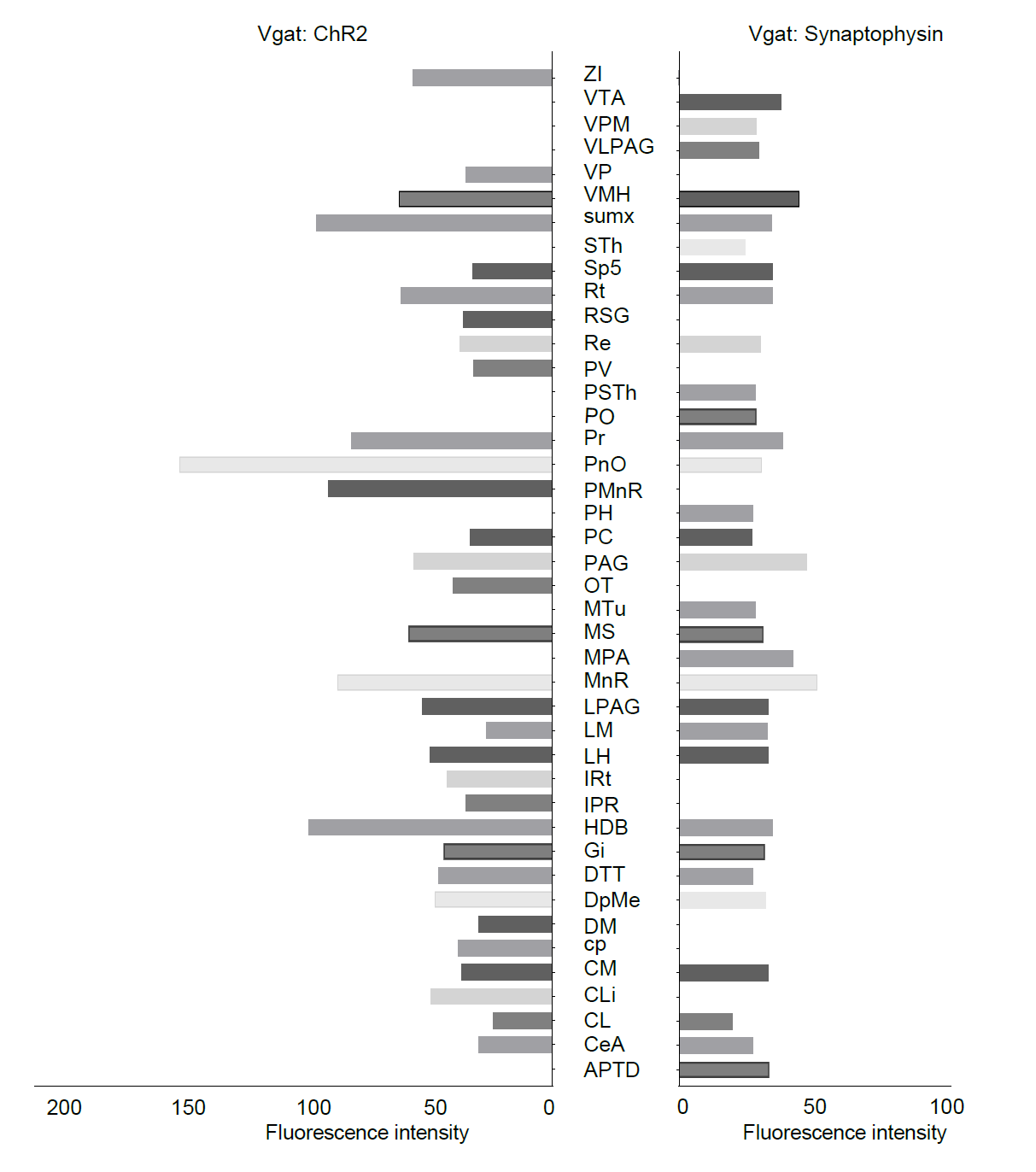
**Figure S10.** Bar graph showing the percentage of each target region that is covered by axonal fibers from LC-GABA neurons (n = 3-6 sections for each region from 3 mice in each group).

CeA, central amygdala nucleus; CL, central lateral thalamic nucleus; CLI, caudal linear nucleus; CM, central medial nucleus of the thalamus; cp, cerebral peduncle; DM, dorsomedial thalamic nucleus; DpMe, deep mesencephalic reticular nucleus; DTT, dorsal tegmental nucleus; Gi, gigantocellular reticular nucleus; HDB, horizontal limb of the diagonal band; IPR, interpeduncular nucleus, rostral subnucleus; IRt, intermediate reticular nucleus; LH, lateral hypothalamus; LM, lateral mammillary nucleus; LPAG, lateral periaqueductal gray; MnR, medial raphe nuclei; MS, medial septum; OT, optic tract nucleus; PAG, periaqueductal gray; PC, paracentral thalamic nucleus; PnO, oral part of the pontine reticular nucleus; Pr, pregeniculate nucleus; Re, reuniens nucleus; RSG, retrosplenial granular cortex; Rt, reticular nucleus; Sp5, spinal trigeminal tract; SUMx, supramammillary nucleus, pars supracapsularis; VMH, ventromedial hypothalamic nucleus; ZI, zona incerta.


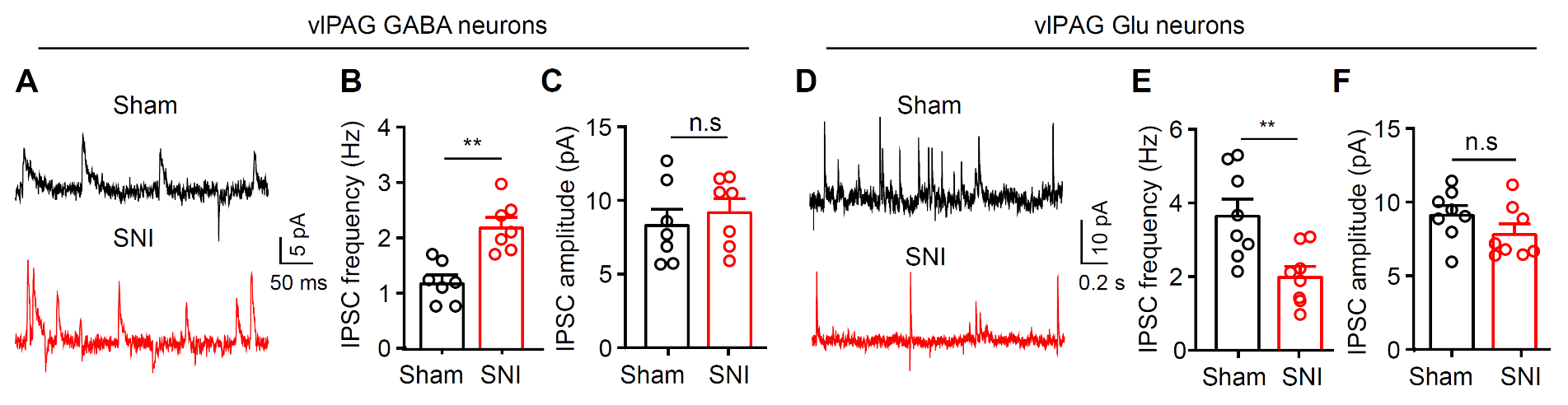
**Figure S11. Comparison of spontaneous inhibitory postsynaptic transmission onto vlPAG GABA and Glu neurons between sham and SNI mice**

**(A-C)** Example traces **(A)** and summary (**B, C)** of spontaneous inhibitory postsynaptic currents (IPSCs) recorded at a holding voltage of -40 mV from mCherry-labeled vlPAG-GABA neurons in sham and SNI mice. **(B)** sIPSC frequency, t = 4.64, P = 0.0006; **(C)** sIPSC amplitude, t = 0.65, P = 0.53; n = 7 mCherry-labeled cells in each group from 3 mice.

**(D-F)** Example traces **(D)** and summary data **(E, F)** of sIPSCs recorded at a holding voltage of -40 mV from mCherry-labeled vlPAG-Glu neurons in sham and SNI mice. (**E)** sIPSC frequency, t = 3.29, P = 0.0053; (**F)** sIPSC amplitude, t = 1.47, P = 0.16; n = 8 mCherry-labeled cells from 3 mice in each group.

‘**’ p < 0.01; ‘ns’ not significant, Two-tailed unpaired *t*-test.


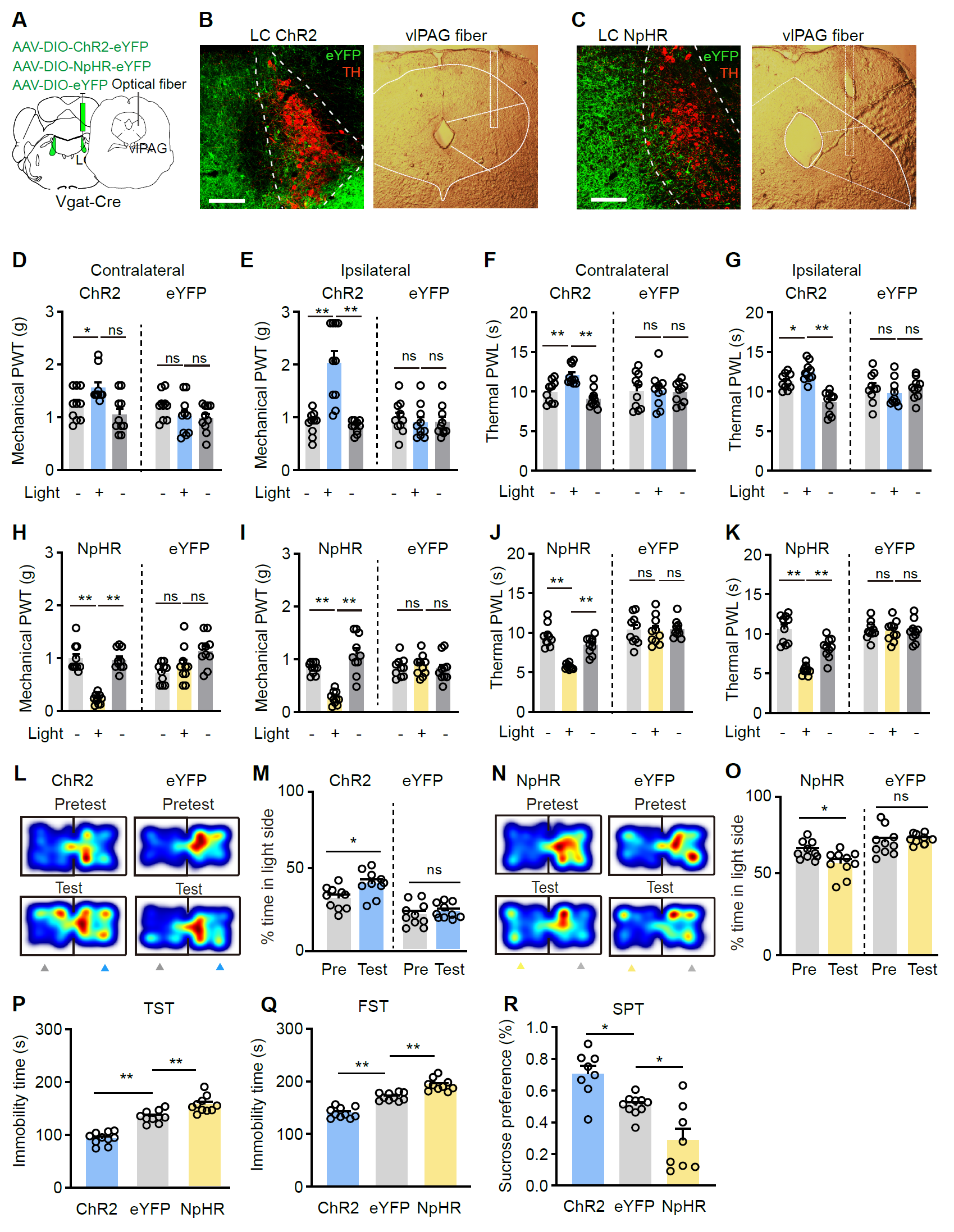
**Figure S12. LC-vlPAG GABAergic projections modulate pain thresholds and depression-like behaviors in naive mice.**

**(A)** Schematic diagram for virus injection and optical fiber placement for selective activation or inhibition of LC-GABAergic axonal terminals in the vlPAG.

**(B)** Example image of an optical fiber placement in the vlPAG in a ChR2 expressing- mouse.

**(C)** Example images of an optical fiber placement in the vlPAG in a NpHR expressing- mouse.

**(D-G)** The effect of blue light illumination on mechanical PWT and thermal PWL in ChR2- and eYFP-mice. **(D)** Light condition: F_(2, 36)_ = 4.98, P = 0.012; **(E)** Light condition: F_(2, 36)_ = 22.77, P < 0.0001. **(F)** Light condition: F_(2, 36)_ = 5.82, P < 0.0001. **(G)** Light condition: F_(2, 36)_ = 16.97, P < 0.0001. n = 10 mice in each group.

**(H-K)** The effect of yellow light illumination on mechanical PWT and thermal PWL in NpHR- and eYFP-mice. **(H)** Light condition: F_(2, 36)_ = 30.97, P < 0.0001. **(I)** Light condition: F_(2, 36)_ = 25.12, P < 0.0001. **(J)** Light condition: F_(2, 36)_ = 21.56, P < 0.0001. **(K)** Light condition: F_(2, 36)_ = 26.69, P < 0.0001. n = 10 mice in each group.

**(L-O)** Example heat maps **(L, N,)** and quantification of % time spent in light paired chamber during precondition and test sessions **(M, O**). **(M)** ChR2 mice, t = 3.23, P = 0.01; eYFP mice, t = 0.93, P = 0.38; **(O)** NpHR mice, t = 3.06, P = 0.014; eYFP mice, t = 0.85, P = 0.42; n = 10 mice in each group.

**(P-R)** Quantification of depression-like behaviors in three groups of mice. **(P)** Immobility time in the TST, F_(2, 27)_ = 57.17, P < 0.0001, n = 10 mice in each group; **(Q)** Immobility time in the FST, F_(2, 27)_ = 74.25, P < 0.0001, n = 10 mice in each group; **(R)** Sucrose preference in the SPT, F_(2, 23)_ = 16.95, P < 0.0001; n = 8 in ChR2 mice, n = 8 in NpHR mice, n =10 in eYFP mice.

*P < 0.05. **P < 0.01; Two-way repeated measures ANOVA with Tukey’s post-hoc analysis for **(D-K)**; Two-tailed paired *t*-test for **(M, O)**; One-way ANOVA with Tukey’s post-hoc analysis for **(P-R)**; Scale bars: 100 μm.


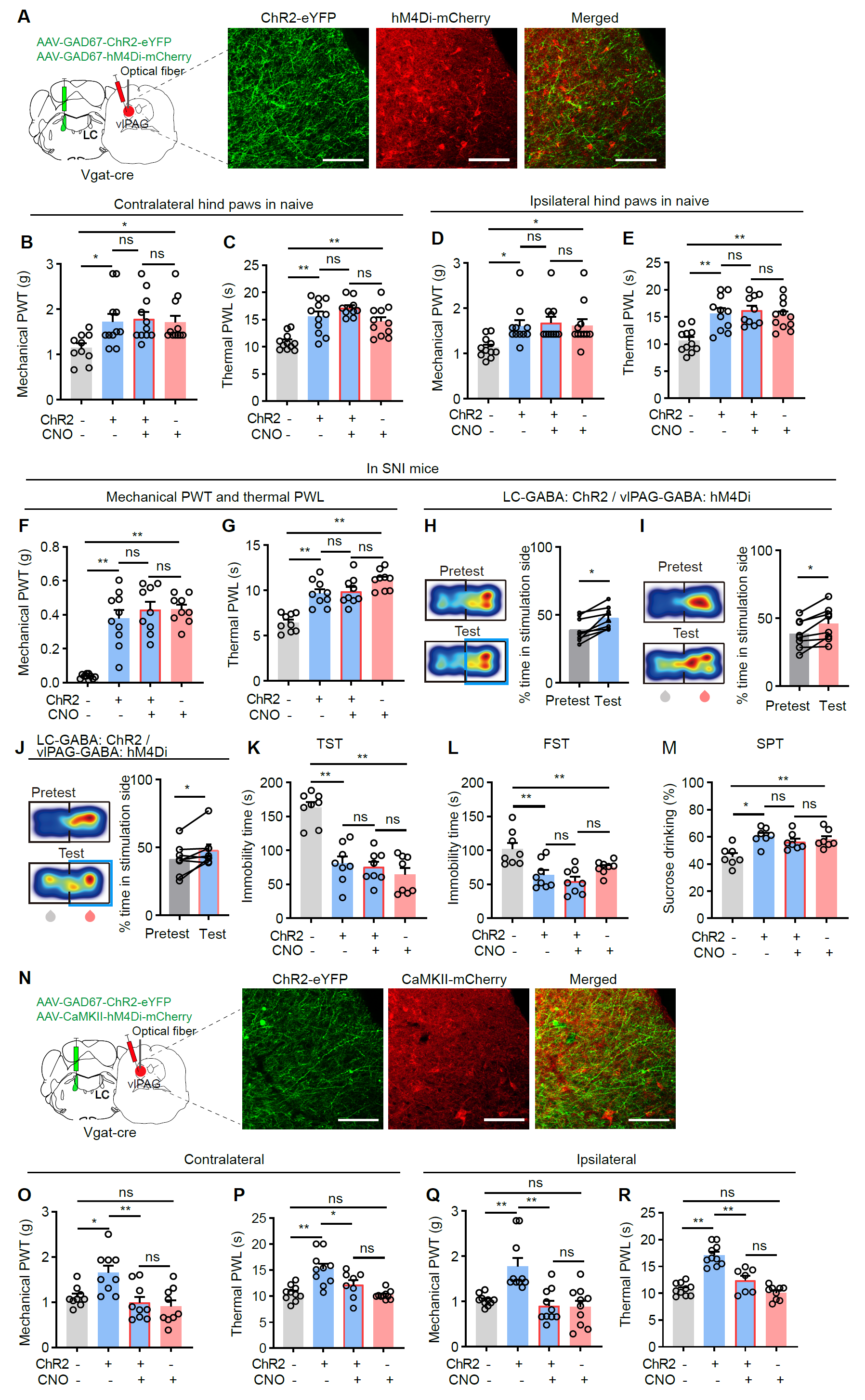
**Figure S13. The roles of GABA and Glu neurons in vlPAG in mediating the behavioral effects of activation of LC^GABA^-PAG pathway**

**(A)** Left panel: Schematic diagram for virus injection and optical fiber placement for selective activation of LC-GABAergic axonal terminals in the vlPAG and chemogenetic inhibition of vlPAG-GABA neurons. Right panel: Example image showing terminals from ChR2-labeled-LC-GABA neurons and hM4Di-mCherry-expressing neurons in the vlPAG.

**(B**-**E)** Pain thresholds on hind paws when the LC^GABA^-vlPAG projection was stimulated by blue light, vlPAG GABA neurons were inhibited by CNO, or these two components were simultaneously modulated by blue light and CNO. Contralateral PWT: F_(3, 39)_ = 3.92, P = 0.016, n >= 10 mice per group. Contralateral PWL: F_(3, 39)_ = 9.73, P < 0.0001, n >= 10 mice per group. **(D)** Ipsilateral PWT: F_(3, 40)_ = 4.34, P = 0.0097, n >= 10 mice per group. **(E)** Ipsilateral PWL: F_(3, 39)_ = 13.4, P < 0.0001, n >= 10 mice per group.

**(K** and **L)** Pain thresholds on the hind paw of SNI surgery side when the LC^GABA^-vlPAG projection was stimulated by blue light, vlPAG GABA neurons were inhibited by CNO, or these two components were simultaneously modulated by blue light and CNO. PWT: F_(3, 34)_ = 27.91, P < 0.001, n >= 9 SNI mice. PWL: F_(3, 34)_ = 22.34, P < 0.0001, n >= 10 SNI mice.

**(F** - **H)** CPP tests. **F**: % time spent in blue light-paired chamber, t = 4.43, P = 0.0022, pretest vs test, n = 9 SNI mice; **G**: % time spent in CNO-paired chamber, t = 3.90, P = 0.0059, n = 8 SNI mice; **H**, % time spent in light + CNO-paired chamber, t = 2.48, P = 0.04, n = 8 SNI mice.

**(I-K)** Depression-like behaviors when the LC^GABA^-vlPAG projection was stimulated by blue light, vlPAG GABA neurons were inhibited by CNO, or these two components were simultaneously modulated by blue light and CNO. **(I)** Immobility time in TST. F_(3, 28)_ = 24.65, P < 0.0001, n = 8 mice in each group. **(J)** Immobility time in the FST. F_(3, 28)_ = 9.07, P = 0.0002, n = 8 mice in each group. **(K)** Sucrose preference in the SPT. F_(3, 24)_ = 24.65, P = 0.0014, n = 7 mice in each group.

**(L)** Left panel: Schematic diagram for virus injection and optical fiber placement for selective activation of LC-GABAergic axonal terminals in the vlPAG and chemogenetic inhibition of vlPAG-Glu neurons. Right panel: Example image showing ChR2 expressing- LC-GABA terminals and hM4Di-mCherry-expressing neurons in vlPAG.

**(M-P)** Pain thresholds on hind paws when the LC^GABA^-vlPAG projection was stimulated by blue light, vlPAG Glu neurons were inhibited by CNO, or these two components were simultaneously modulated by blue light and CNO. **(M)** Contralateral PWT: F_(3, 39)_ = 7.09, P = 0.009, n = 9 mice per group. **(N)** Contralateral PWL: F_(3, 34)_ = 11.34, P < 0.0001, n = 8-10 mice per group. **(O)** Ipsilateral PWT: F_(3, 40)_ = 10.84, P = 0.0097, n = 10 mice per group. **(P)** Ipsilateral PWL: F_(3, 32)_ = 33.92, P < 0.0001, n = 8-10 mice per group.

*P < 0.05. **P < 0.01; One-way ANOVA with Tukey’s post-hoc analysis for **(B-L, I-K, M-P)**; Two-tailed paired *t*-test for **(F-H)**; Scale bars: 100 μm.
